# Sirt4 Deficiency Promotes Cardiomyocyte Proliferation and Cardiac Repair

**DOI:** 10.1111/jcmm.70741

**Published:** 2025-08-21

**Authors:** Weijing Liu, Jie Feng, Yuan Zhang, Yanyan Hao, Jiajun Zhong, Xinchang Liu, Dongcheng Cai, Haorui Liu, Lina Bai, Miaoqing Hu, Hong Lian, Yu Nie, Houzao Chen, Yuyao Wang

**Affiliations:** ^1^ State Key Laboratory of Cardiovascular Disease, Fuwai Hospital, National Center for Cardiovascular Disease Chinese Academy of Medical Sciences and Peking Union Medical College Beijing China; ^2^ Department of Biochemistry and Molecular Biology Shanxi Medical University Taiyuan China; ^3^ National Health Commission Key Laboratory of Cardiovascular Regenerative Medicine, Central China Subcenter of National Center for Cardiovascular Disease, Henan Cardiovascular Disease Center, Fuwai Center‐China Cardiovascular Hospital Central China Fuwai Hospital of Zhengzhou University Zhengzhou China; ^4^ National Health Commission Key Laboratory of Cardiovascular Regenerative Medicine, Fuwai Central‐China Hospital, Central China Branch of National Center for Cardiovascular Diseases Zhengzhou University Zhengzhou China; ^5^ Department of Physiology & Pathophysiology, School of Basic Medical Sciences Capital Medical University Beijing China; ^6^ Department of Cardiology and Institute of Vascular Medicine Peking University Third Hospital Beijing China; ^7^ State Key Laboratory of Vascular Homeostasis and Remodeling Peking University Beijing China; ^8^ NHC Key Laboratory of Cardiovascular Molecular Biology and Regulatory Peptides Peking University Beijing China; ^9^ Beijing Key Laboratory of Cardiovascular Receptors Research Beijing China; ^10^ Shenzhen Key Laboratory of Cardiovascular Disease Fuwai Hospital Chinese Academy of Medical Sciences Shenzhen China; ^11^ State Key Laboratory of Medical Molecular Biology, Department of Biochemistry and Molecular Biology, Institute of Basic Medical Sciences Chinese Academy of Medical Sciences &Peking Union Medical College Beijing China

**Keywords:** cardiomyocyte proliferation, heart regeneration, oxidative DNA damage, Sirt4

## Abstract

The mammalian heart exhibits transient but remarkable regenerative capacity during the early postnatal period, after which most cardiomyocytes exit the cell cycle. While the sirtuin family is well‐established as regulators of cell cycle progression, its specific role in cardiomyocyte proliferation and cardiac regeneration remains unclear. In this study, we found that Sirt4 expression increased during postnatal heart development. Adenovirus‐mediated *Sirt4* overexpression in vitro inhibited cardiomyocyte proliferation by inducing oxidative DNA damage. Moreover, cardiomyocyte‐specific *Sirt4* overexpression in vivo suppressed cardiomyocyte proliferation and impaired neonatal heart regeneration. Using *Sirt4*‐knockout mice, we found that Sirt4 deficiency promoted cardiomyocyte proliferation and extended the heart regeneration window. Furthermore, *Sirt4* deficiency improved cardiac function and reduced myocardial fibrosis after ischaemia–reperfusion injury in adult mice. These findings establish Sirt4 as a critical regulator of cardiomyocyte proliferation and cardiac repair, suggesting that targeted Sirt4 inhibition may represent a promising therapeutic strategy for ischaemic heart diseases.

## 1 Introduction

Heart failure resulting from cardiomyocyte loss or dysfunction remains a leading cause of human morbidity and mortality [1, 2], highlighting the urgent need for innovative heart regeneration strategies. The adult heart faces multiple biological barriers to regeneration, with the inability of adult cardiomyocytes to proliferate being paramount [3]. In contrast, neonatal mouse hearts retain regenerative capacity shortly after birth, as cardiomyocytes maintain residual proliferative potential from embryonic development [4, 5, 6, 7]. The mammalian fetal heart resides in relatively hypoxic environments [8]. However, the transition from embryonic‐ to postnatal circulation soon after birth drastically changes the oxygenation state of cardiomyocytes [9, 10], which results in increased mitochondrial reactive oxygen species (ROS) production [8, 11]. Notably, ROS‐mediated activation of the DNA damage response (DDR) has been identified as a crucial molecular mechanism that drives the cell cycle withdrawal of postnatal cardiomyocytes, thereby restricting cardiac regenerative capacity [8, 12, 13].

Sirtuins are highly conserved NAD^+^‐dependent deacetylases and ADP‐ribosyltransferases [14, 15, 16]. Mammals express seven sirtuin homologues (Sirt1–7) that regulate diverse cellular processes including stress resistance, senescence, tumorigenesis, and neurodegenerative disorders [17, 18]. Considering mitochondrial ROS production, Sirt3, Sirt4 and Sirt5 have garnered attention for their specific localisation in mitochondria. Cardiac‐specific expression of Sirt3 protects against hypertrophic stimuli by enhancing antioxidant activity, thereby reducing the cellular ROS levels [19]. Sirt4 deficiency confers resistance to angiotensin II‐induced hypertrophy and fibrosis [20]. Sirt5 interacts with Bcl‐xl to protect cardiomyocytes from oxidative stress [21]. Although sirtuins have been studied in cardiovascular diseases, their roles in heart regeneration remain unexplored.

In this study, we found that among sirtuin family members, *Sirt4* and *Sirt5* expression increased with age and decreased post‐myocardial infarction (MI) in neonatal mice. The overexpression of *Sirt4*, but not Sirt5, inhibited primary neonatal cardiomyocyte proliferation, whereas *Sirt4* knockdown enhanced proliferative capacity in cardiomyocytes. Cardiomyocyte‐specific *Sirt4* overexpression in vivo impaired neonatal heart regeneration following apex resection. Conversely, Sirt4 deficiency enhanced cardiomyocyte proliferation and improved cardiac repair in both juvenile and adult mice following myocardial injury. Mechanically, Sirt4 induced ROS accumulation and subsequent oxidative DNA damage, thereby inhibiting cardiomyocyte proliferation. Our findings establish Sirt4 as a critical negative regulator of cardiomyocyte proliferation, suggesting its potential as a therapeutic target for cardiac repair following myocardial injury.

## 2 Materials and Methods

### 2.1 Mice

For the wild type (WT) experiments, *C57BL*/*6* mice procured from Vital River Laboratory Animal Technology Co. Ltd. (Beijing, China) were used. The *Sirt4*‐knockout (KO) mice, originally on a 129sv background (stock number: 012756), were acquired from Jackson Laboratories. These mice were backcrossed with *C57BL*/*6* mice over six generations to establish *Sirt4‐*KO mice on a *C57BL*/*6* background. *Sirt4* transgenic mice were generated following the previously established protocols [20]. All animal experiments were conducted within the SPF barrier facilities at Fuwai Hospital, Chinese Academy of Medical Sciences. Mice were euthanised using the cervical dislocation method. All animal procedures were performed in compliance with the Guide for the Care and Use of Laboratory Animals and received approval from the Institutional Animal Care and Use Committee at Fuwai Hospital, Chinese Academy of Medical Sciences (FW‐2019‐0005). The sex and body weight of the *Sirt4*‐Tg and *Sirt4*‐KO mice used in the experiment were listed in Table S1.

### 2.2 Genotyping

The genotyping of *Sirt4*‐Tg and *Sirt4*‐KO mice was performed through PCR analysis. DNA was extracted by lysing the mouse tail with 50 mM NaOH at 95°C for 30 min. The PCR system was configured and performed according to the reagent instructions (P213‐01, Vazyme). The primers were listed in Table S2.

### 2.3 Cell Culture

Primary cardiomyocytes were isolated from neonatal WT mice using the Neonatal Heart Dissociation Kit (Miltenyi Biotec, 130‐098‐373) following the manufacturer's instructions. The isolated cardiomyocytes were cultured in Dulbecco's Modified Eagle's Medium (DMEM) supplemented with 10% fetal bovine serum (FBS) under conditions of 37°C and 5% CO_2_. The cardiomyocytes were seeded in 96‐well plates (5 × 10^4^ cells per well, for cardiomyocyte proliferation analysis), 12‐well plates (5 × 10^5^ cells per well, for RNA extraction), and 6‐well plates (1 × 10^6^ cells per well, for western blot analysis). After 24 h of cell attachment, cardiomyocytes were subjected to various experimental treatments based on the specific study design.

### 2.4 Adenovirus‐Mediated Gene Overexpression

The recombinant adenoviruses were commercially obtained from HanBio Biotechnology Co. Ltd. (Shanghai, China), a reputable provider with ISO 9001 certification for viral vector production. Cardiomyocytes were incubated with adenoviruses for 48 h for gene overexpression.

### 2.5 RNA Interference‐Mediated Gene Silencing

For RNA interference‐mediated gene silencing in vitro, we used specific siRNA targeting *Sirt4*. The isolated primary cardiomyocytes were transfected with siRNA using Lipofectamine 3000 for 48 h. The siRNA sequence was as follows: sense, 5′‐GAUGUCCAAAGGCUGGAAATT‐3′, antisense, 5′‐UUUCCAGCCUUUGGACAUCTT‐3′.

### 2.6 Apical Resection

The apical resection (AR) procedure on postnatal Day 1 (P1) mice was executed following established protocols [22]. Anaesthesia was induced by subjecting neonatal mice to hypothermia on ice for 2–3 min. Subsequently, left parasternal thoracotomy was performed at the fourth intercostal space via blunt dissection after a transverse skin incision. Expose the heart and excise 10%–15% of the heart apex using iridectomy scissors. Post‐resection, the thoracic wall and skin were sutured with 8–0 non‐absorbable silk suture. The neonates were carefully placed on a 37°C warming platform to facilitate recovery, after which they were returned to their cages.

### 2.7 Myocardial Infarction

Myocardial infarction (MI) procedure on P7 mice was executed following established protocols [23, 24]. P7 mice were anaesthetised using hypothermia on ice for 3 min and positioned in right lateral decubitus on the operating platform. A thoracotomy was performed at the fourth intercostal space, and the left anterior descending (LAD) coronary artery was ligated with 8–0 non‐absorbable silk suture to create ischaemia in the left ventricle. The thoracic wall and skin incisions were sutured with 7–0 non‐absorbable silk suture. Postoperative recovery was facilitated by placing the mice on a 37°C warming blanket until they regained consciousness, after which they were returned to their cages. The same procedures were performed without ligating the LAD in the sham group.

### 2.8 Ischemia–Reperfusion

Ischemia–reperfusion (I‐R) procedure on adult mice was performed following established protocols [25]. Mice were anaesthetised with 4.5% isoflurane, then maintained on a ventilator (225 strokes/min, 250 μL stroke volume) delivering oxygen with 1.5% isoflurane. A left thoracotomy was performed at the fourth intercostal space, and the LAD coronary artery was ligated to induce ischaemia in the left ventricle. After 30 min of occlusion, the ligature was removed. The thoracic wall and skin incisions were sutured with 6–0 non‐absorbable sutures. The entire surgical procedure was carried out on a 37°C warming blanket to maintain body temperature. Mice were returned to cages upon regaining consciousness. The same procedures were performed without ligating the LAD in the sham group.

### 2.9 Nucleation Measurement

Cardiomyocytes were isolated from mouse hearts using collagenase II (Worthington, LS004176) and collagenase IV (Worthington, LS004188) according to a previously established protocol [26]. The cardiomyocyte suspension was thinly smeared onto glass coverslips and air‐dried. Immunofluorescent staining was performed with α‐actinin (Abcam, ab9465, 1:200) and DAPI (Sigma, F6057, 1:100). Fluorescence was observed under a ZEISS LSM800 confocal laser scanning microscope (Carl Zeiss Inc., Jena, Germany). Cardiomyocytes with mononuclear, binucleate and multinucleate forms were counted. At least 300 cardiomyocytes per sample were analysed.

### 2.10 qRT–PCR

Total RNA was extracted using TRIzol reagent (Invitrogen, 15596026CN). cDNA was synthesised using PrimeScript RT Master Mix (Takara, RR036A). qRT–PCR was performed with SYBR Green qPCR Master Mix (Applied Biosystems, 4309155) on the QuantStudio 5 RT–PCR System (Applied Biosystems). Gene expression was assessed and quantified using the ΔΔCt method. The primers for the reactions were listed in Table S3.

### 2.11 Immunofluorescence

Mice were euthanised via cervical dislocation. The harvested hearts were fixed in 4% paraformaldehyde at room temperature for 48 h, then dehydrated in an ethanol and xylene series, and finally paraffin‐embedded. The heart paraffin sections undergo antigen retrieval using EDTA antigen retrieval buffer, followed by permeabilisation and blocking with 0.3% Triton X‐100 and 20% donkey serum for 1 h at room temperature. Sections were incubated with primary antibodies overnight at 4°C, washed five times with PBS, and then incubated with fluorescence‐labelled secondary antibody for 1 h at room temperature in the dark. For cardiomyocytes, antigen retrieval was omitted; instead, cells were fixed in 4% paraformaldehyde for 15 min before permeabilisation and blocking. DAPI was used for nuclear staining. Fluorescence was observed under a Zeiss LSM800 confocal laser scanning microscope (Carl Zeiss Inc., Jena, Germany). The cardiomyocytes proliferation rate was quantified through high‐content analysis (Perkin Elmer, Massachusetts, USA) [27, 28]. For primary mouse cardiomyocytes cultured in 96‐well plates, we captured 20× magnification images covering each well (*n* = 25 fields/well). For heart sections, we captured 20× magnification images covering the entire heart. The primary antibodies and secondary antibodies were listed in Table S4.

### 2.12 Re‐Analysis of Published Datasets

Previously published bulk RNA‐sequencing (RNA‐seq) datasets from Yang Huijun et al. [29] were reanalysed in this study (PRJNA783509). The datasets contained data from whole heart lysates from P0 and P60. Differential expression analysis was performed with Hiplot.

### 2.13 RNA‐Seq

Sham and MI operations were performed on neonatal *C57BL*/*6J* mice at P1. Hearts were harvested at 1 day post operation and total RNA was extracted using TRIzol reagent (Invitrogen, 15596026CN). RNA library preparation and sequencing were carried out by Novogene (Beijing, China). FastQC (Version 0.11.9) was used to perform a quality assessment on all fastq files. Low‐quality segments were trimmed using TrimGalore (Version 0.39). Next, the clean reads were aligned to the mouse reference genome (mm10) using HISAT2 (Version: 2.1.0). Gene expression was quantified with featureCounts (Version 1.5.0). DESeq2 (Version: 1.34.0) was applied to raw counts transcriptome data to determine differentially expressed genes. The raw RNA‐seq data have been deposited in the NCBI BioProject database under accession number PRJNA1159548.

### 2.14 Western Blot

The heart tissue and cardiomyocytes were lysed in RIPA lysis buffer containing 1 mM phenylmethylsulfonyl fluoride (PMSF) (Beyotime Institute of Biotechnology) for 40 min. The tissue or cell suspension was centrifuged at 12,000 rpm for 15 min at 4°C, following which the supernatant was collected as the protein extract. Protein was loaded onto 4%–12% Bis‐Tris Gels for separation and then transferred to a PVDF membrane (Millipore). After blocking with QuickBlock Blocking Buffer (Beyotime Institute of Biotechnology) for 1 h, the membranes were incubated with the primary antibodies at 4°C overnight. Then, the membranes were washed three times with TBST and incubated with the secondary antibody for 1 h at room temperature. Protein signals were detected using Pierce ECL western blot Substrate (Thermo Fisher Scientific, 32209). The primary antibodies and secondary antibodies were listed in Table S4.

### 2.15 ROS Measurement

ROS measurement was performed following established protocol [30]. The cardiomyocytes were incubated with oxidation‐sensitive probe DHE (10 μM, C1300‐2, Applygen, China) at 37°C in dark for 30 min. Then the cardiomyocytes were washed twice with cold PBS and resuspended in the PBS for analysis of intracellular ROS by flow cytometer.

### 2.16 Echocardiography

For cardiac function measurement, mice were assessed using echocardiography with a Vevo2100 micro‐ultrasound system (Vevo 2100 Imaging System, Visual Sonics, Toronto, Canada). All the mice were initially anaesthetised with 2%–3% isoflurane, and then maintained at 1%–2% during the process of echocardiographic assessment. The parasternal long‐axis view was used to obtain echocardiographic M‐mode images. Left ventricular end‐diastolic wall thickness and the ejection fraction and fractional shortening were measured through isual Sonics Vevo imaging software from M‐mode image. At least 5 cardiac cycles of echocardiography analysis are required for each mouse.

### 2.17 Transmission Electron Microscopy

Cardiomyocytes were fixed using 1% glutaraldehyde. Sectioning and staining for transmission electron microscopy (TEM) were conducted at the Cell Biology Platform of Tsinghua University. Mitochondrial structural images were captured using a transmission electron microscope (JEM‐1400flash, Japan). To analyse mitochondrial morphology, at least 10 high‐resolution images of mitochondria were captured at 40000 × magnification and analysed using ImageJ.

### 2.18 Wheat Germ Agglutinin (WGA) Staining

Heart sections were incubated with WGA conjugated to Alexa Fluor 647 (Invitrogen, W32466, 1:200) for 1.5 h at room temperature, followed by PBS washing. Fluorescence was observed using a Zeiss LSM800 confocal microscope (Carl Zeiss Inc., Jena, Germany). For cross‐sectional cell size quantification, three hearts per group were analysed at three different views (left and right ventricles, septum) at 40× magnification. ImageJ was used to measure the size of round CMs containing a nucleus, with at least 500 cells per sample quantified.

### 2.19 Histological Examination

Cardiac infarct size and phenotypes were examined using Masson's trichrome staining (Sigma, HT15) and Haematoxylin–Eosin staining according to standard procedures. To assess infarct size, we used ImageJ software to calculate the infarct area and total heart area. Scar (blue) and healthy tissue (red) were measured on transverse sections across four levels (100 μm apart, starting just below the ligation) of the left ventricle in MI hearts. At least four tissue levels per heart were measured, and infarct size was calculated as the ratio of infarct area to the total area of the four levels.

### 2.20 Statistical Analysis

All data are presented as the mean ± standard error of the mean (SEM). Differences between the two groups were compared using an unpaired Student's *t*‐test. For comparison of multiple groups, one‐way ANOVA and two‐way ANOVA were used. The Bonferroni's multiple comparisons test was used for the post hoc analysis of ANOVA results. All statistical analyses were performed using GraphPad Prism 8 software. The results with *p*‐values of less than 0.05 were considered statistically significant.

## 3 Results

### 3.1 Sirt4 Overexpression Inhibits Cardiomyocyte Proliferation

**FIGURE 1:**
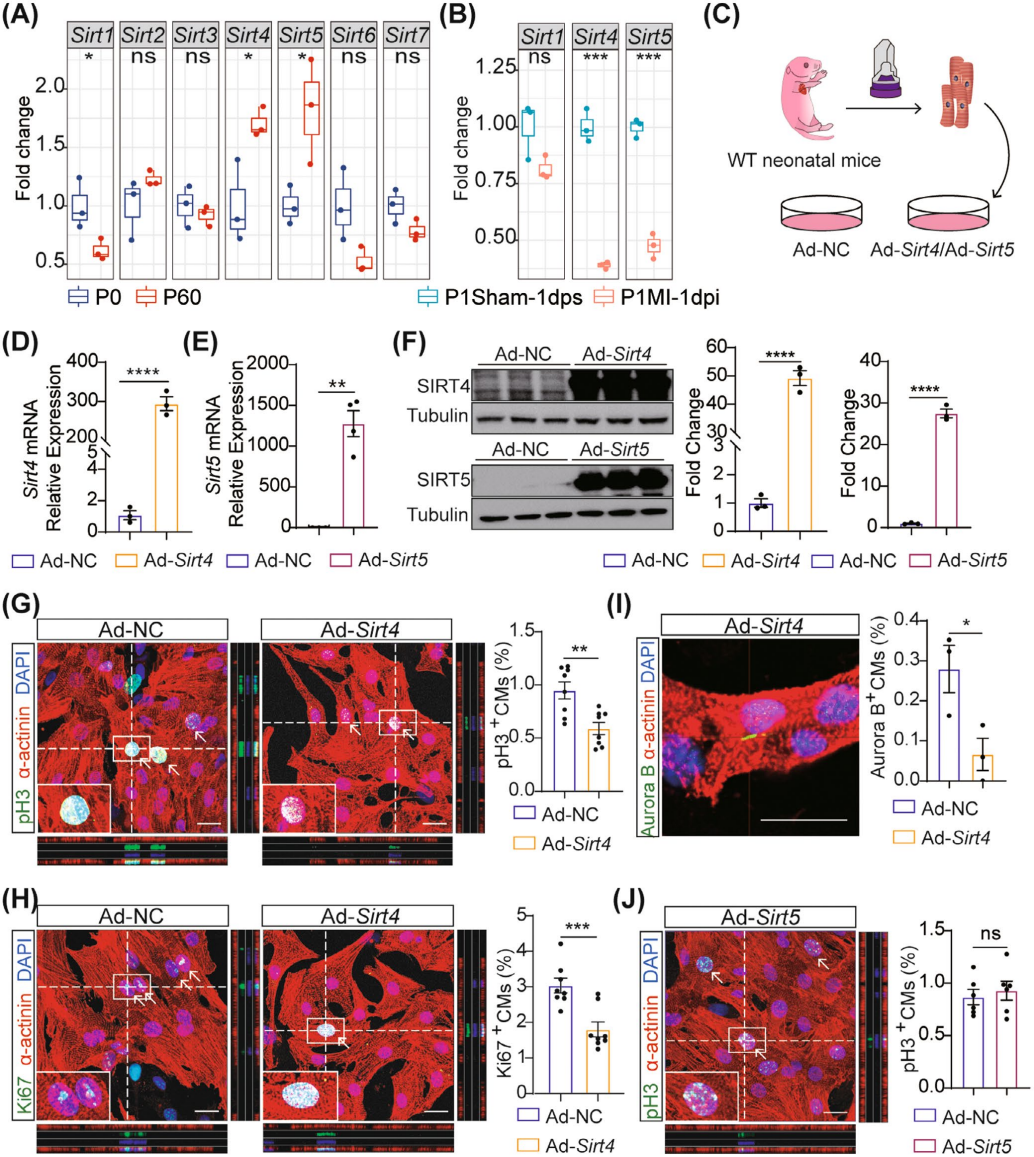
Sirt4 overexpression inhibits cardiomyocyte proliferation in vitro. (A, B) Boxplot showing RNA expression of Sirtuins genes in mouse heart samples during heart development (A) or subjected to myocardial infarction (MI) (B) based on RNA‐sequencing data (*n* = 3 biological replicates). P0, postnatal Day 0; P60, postnatal Day 60; dps, day post sham; dpi, day post infarction. (C) Schematic representation of the experiment design. Neonatal mouse cardiomyocytes isolated from P1 mice (P1‐NMCMs) were infected with Ad‐*Sirt4* and Ad‐*Sirt5* for 48 h, with Ad‐NC treatment was the controls. WT, wild type; Ad‐NC, adenovirus (Ad) harbouring a vector control; Ad‐*Sirt4*, adenovirus (Ad) harbouring *Sirt4*; Ad‐*Sirt5*, adenovirus (Ad) harbouring *Sirt5*. (D, E) Quantitative real‐time polymerase chain reaction (qRT‐PCR) analysis of *Sirt4* and *Sirt5* expression in P1 neonatal mice cardiomyocytes (P1‐NMCMs) infected with Ad‐NC and Ad‐*Sirt4*, Ad‐*Sirt5* for 48 h (*n* = 3 biological replicates). (F) Western blot analysis of SIRT4 and SIRT5 expression in P1‐NMCMs infected with Ad‐NC and Ad‐*Sirt4*, or Ad‐*Sirt5* for 48 h (*n* = 3 biological replicates). (G–I) Immunofluorescence analysis of the proliferation of P1‐NMCMs infected with Ad‐NC and Ad‐*Sirt4* for 48 h (*N* = 8 biological replicates for pH 3^+^ and Ki67^+^ CMs, *n* = 3 biological replicates for Aurora B^+^ CMs). White arrows indicate pH 3^+^, Ki67^+^ or Aurora B^+^ CMs. Scale bars, 20 μm. (J) Immunofluorescence analysis of the proliferation of P1‐NMCMs infected with Ad‐NC and Ad‐*Sirt5* for 48 h (*n* = 6 biological replicates). White arrows indicate pH 3^+^ CMs. Scale bars, 20 μm. Data are mean ± SEM; *p < 0.05, **p < 0.01, ***p < 0.001, ****p < 0.0001; ns, not significance; unpaired two‐tailed *t*‐tests (A, B, D–J).

To investigate the roles of sirtuin family members in heart regeneration, we first analysed the expression of Sirt1–7 in mouse hearts at postnatal Day 0 (P0) and P60 based on our previous RNA‐sequencing (RNA‐Seq) data [29]. We found that Sirt1 expression decreased with age, while Sirt4 and Sirt5 levels increased, inversely correlating with cell cycle gene expression (Figure 1A, Figure S1). Other sirtuins showed no significant age‐dependent changes (Figure 1A). We then established a myocardial infarction (MI) model in neonatal P1 mice and performed RNA‐Seq on the heart tissues collected at 1 day post‐MI (dpi). The results showed no significant change in Sirt1 expression, whereas Sirt4 and Sirt5 expression significantly decreased post‐injury (Figure 1B), suggesting their involvement in heart regeneration after myocardial injury.

Since cardiomyocyte proliferation drives heart regeneration [23, 27, 31, 32, 33, 34, 35, 36], we examined the roles of Sirt4 and Sirt5 in cardiomyocyte proliferation. We purchased adenovirus for *Sirt4* and *Sirt5* overexpression (Ad‐*Sirt4* or Ad‐*Sirt5*); the detailed plasmid map can be found in Figure S2. To determine the optimal titre for transduction, we isolated P1 neonatal mouse cardiomyocytes (P1‐NMCMs) and treated them with adenoviruses at three different multiplicities of infection (MOI) for 48 h (MOI: 50, 100, and 200), followed by quantitative assessment of transduction efficiency through GFP‐positive cell counting. Our results revealed a dose‐dependent increase in the percentage of GFP‐positive cardiomyocytes, with a MOI of 100 achieving a high efficiency (80% GFP‐positive cells). Therefore, we selected a MOI of 100 for subsequent transduction experiments (Figure 1C, Figure S3). Quantitative real‐time polymerase chain reaction (qRT‐PCR) and western blot showed increased mRNA and protein levels of Sirt4 and Sirt5 in cardiomyocytes after Ad‐*Sirt4* and Ad‐*Sirt5* infection, respectively (Figure 1D–F, Figure S4). Immunostaining for α‐actinin with Ki67 and phosphorylated histone H3 Ser10 (pH 3) was used to detect cardiomyocyte cell cycle entry and karyokinesis [8, 26, 27, 37, 38, 39] (Figure 1H). The results showed that Sirt4 overexpression significantly reduced the number of Ki67‐ and pH 3‐positive cardiomyocytes. What's more, the localization of the cytokinesis marker Aurora B kinase to the cardiomyocyte cleavage furrow was significantly reduced in Sirt4‐overexpressed cardiomyocytes (Figure 1I), demonstrating its role in inhibiting cardiomyocyte proliferation. However, no obvious changes in cardiomyocyte proliferation were observed in Sirt5‐overexpressed cardiomyocytes (Figure 1J). Furthermore, we utilised siRNA to knock down *Sirt4* in P1‐NMCMs and found that *Sirt4* deficiency promoted cardiomyocyte proliferation (Figure S5A–D). Collectively, these findings suggest that Sirt4 may modulate heart regeneration through cardiomyocyte proliferation.

### 3.2 Ectopically Expressed Sirt4 Impairs Neonatal Heart Regeneration

**FIGURE 2:**
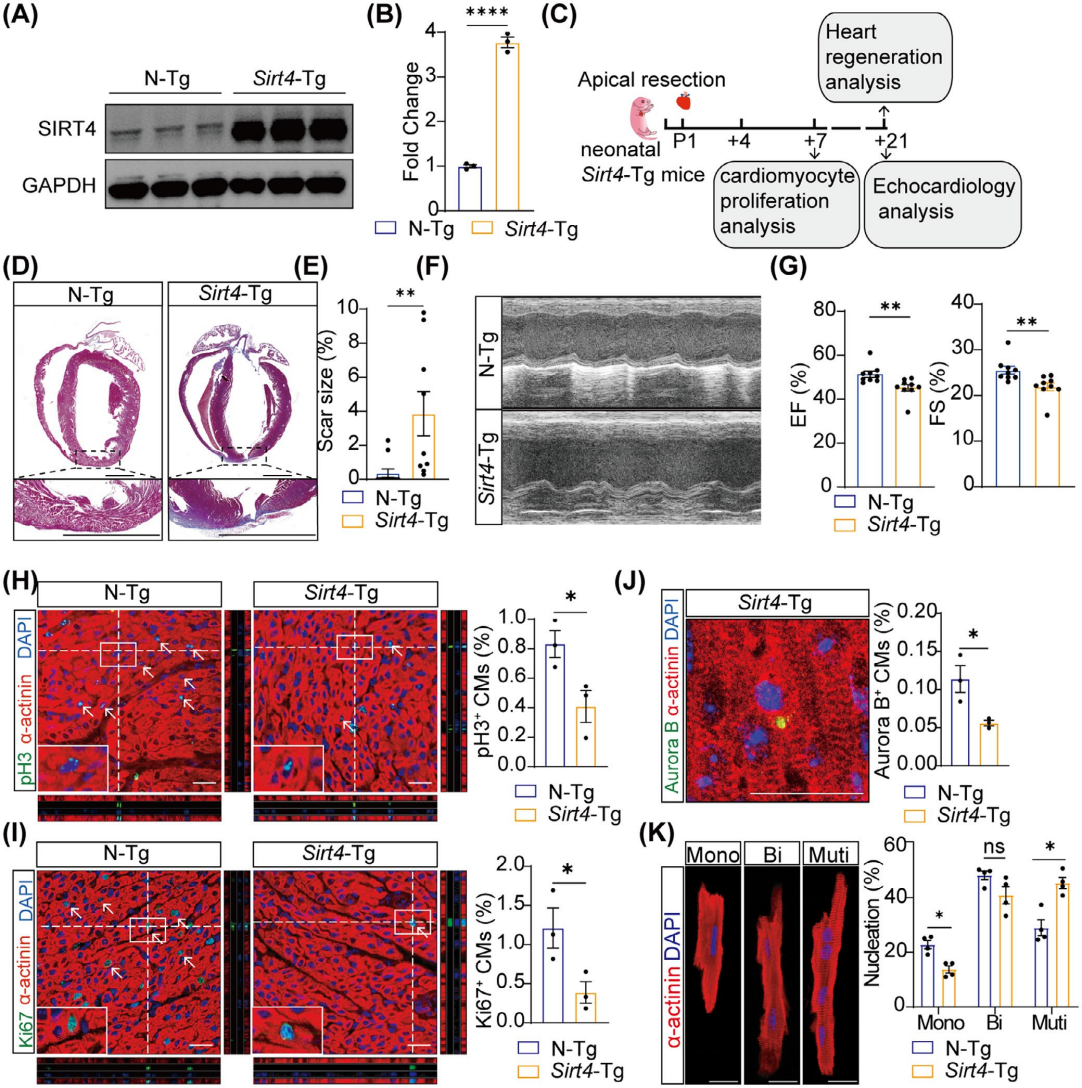
Ectopically expressed Sirt4 impairs heart regeneration in neonatal mice. (A, B) Western blot analysis (A) and quantification (B) of SIRT4 expression in N‐Tg and *Sirt4*‐Tg mice at 7 dpr (*n* = 3 biological replicates). N‐Tg, negative transgenic; *Sirt4*‐Tg, Sirt4 transgenic. (C) Schematic representation of the experiment design. Apical resection was performed on postnatal Day 1 (P1) mice. Hearts were harvested for cardiomyocytes (CMs) proliferation analysis at 7‐day post resection (dpr) and for myocardial fibrosis and echocardiology analysis at 21 dpr. (D, E) Representative Masson trichrome staining of heart sections and the quantification of fibrosis area proportion of N‐Tg and *Sirt4*‐Tg mice at 21 dpr (*n* = 9 mice). Scale bars, 400 μm. (F) Representative images of M‐Mode echocardiographic assessment. (G) The ejection fraction (EF) and fractional shortening (FS) of the left ventricle in N‐Tg and *Sirt4*‐Tg mice at 21 dpr (*n* = 9 mice). (H–J) Immunofluorescence analysis of CM proliferation in heart sections at 7 dpr (*n* = 3 mice). White arrows indicate pH 3^+^ or Ki67^+^, Aurora B^+^ CMs. Scale bars, 20 μm. (K) Immunofluorescence images and quantification of mononucleated, binucleated, and multinucleated CMs isolated at 14 dpr from N‐Tg and *Sirt4*‐Tg mice (*n* = 4 mice). Scale bars, 20 μm. Data are mean ± SEM; *p < 0.05, **p < 0.01, ****p < 0.0001; ns, not significance; unpaired two‐tailed *t*‐tests (B, E, G–J); one‐way ANOVA (K).

To explore whether Sirt4 affected neonatal heart regeneration, we introduced cardiomyocyte‐specific *Sirt4* transgene (*Sirt4*‐Tg) mice and confirmed their genetic backgrounds by PCR‐based genotyping (Figure S6A) [20]. qRT‐PCR and Western blot showed that Sirt4 expression was increased in the hearts of *Sirt4*‐Tg mice compared to that of negative transgene (N‐Tg) mice (Figure 2A,B, Figure S6B,C). Haematoxylin–Eosin (H&E) staining showed no significant morphological differences between N‐Tg and *Sirt4*‐Tg mice at P1 (Figure S6D). We performed apical resection (AR) operation [22] on P1 *Sirt4*‐Tg mice and evaluated the effects of Sirt4 overexpression on cardiomyocyte proliferation and heart regeneration (Figure 2C). A decreased trend in survival rate was observed in *Sirt4*‐Tg mice compared to N‐Tg mice (Figure S6E). Masson's trichrome staining showed larger scar size in *Sirt4*‐Tg hearts at 21‐day post resection (dpr), compared to that of N‐Tg mice (Figure 2D,E, Figure S7). Echocardiography showed that *Sirt4*‐Tg mice exhibited aggravated cardiac dysfunction after AR, with reduced ejection fraction (EF) and fractional shortening (FS) as well as increased E/e’ ratio at 21 dpr (Figure 2F,G, Figure S8A). LV end‐systolic diameter (LVESD), LV end‐diastolic diameter (LVEDD) and LV posterior wall thickness at end‐diastole (LVPW) remained unchanged in *Sirt4*‐Tg mice at 21 dpr (Figure S8B–D). Heart rate was not altered by *Sirt4*‐Tg (Figure S8E). Moreover, cardiomyocyte proliferation was significantly inhibited by Sirt4 overexpression, as evidenced by staining for α‐actinin with pH 3, Ki67 and Aurora B at 7 dpr (Figure 2H–J). Immunostaining of α‐actinin and DAPI showed that *Sirt4*‐Tg mice displayed a decreased fraction of mononucleated cardiomyocytes at 14 dpr (Figure 2K). These results reinforce that Sirt4 overexpression inhibits cardiomyocyte proliferation and neonatal heart regeneration.

### 3.3 Sirt4 Overexpression Induces Abnormal Mitochondrial Structure and Oxidative DNA Damage in Cardiomyocytes

**FIGURE 3:**
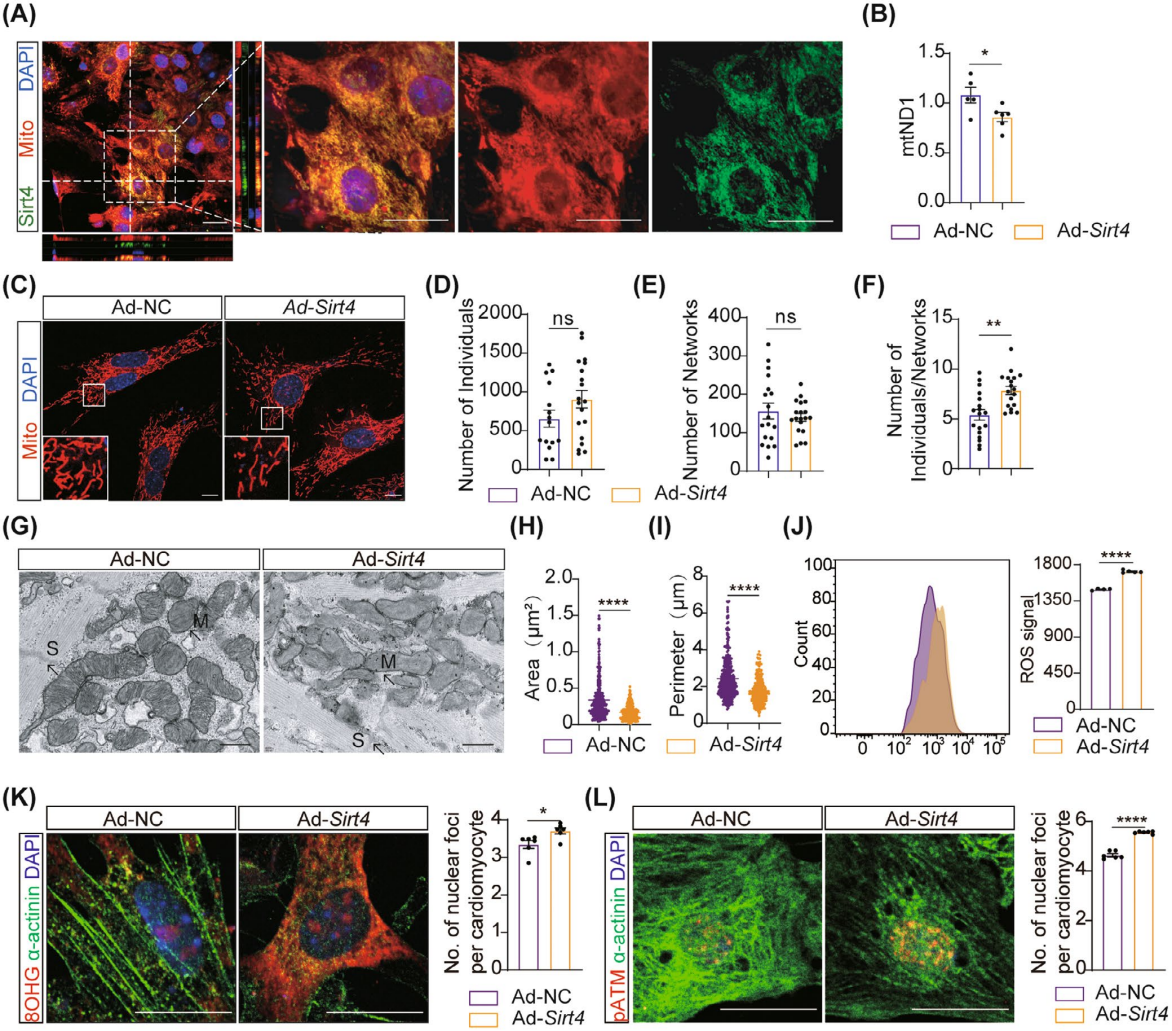
Sirt4 overexpression activates oxidative DNA damage response in cardiomyocytes. (A) Immunofluorescence analysis of Sirt4 location in primary cardiomyocytes isolated from P1 mice (P1‐NMCMs) infected with Ad‐*Sirt4* for 48 h. Mito, mitochondria. Scale bars, 10 μm. (B) Quantitative real‐time polymerase chain reaction (qRT‐PCR) analysis of mitochondrial DNA content using NADH dehydrogenase subunit 1 (mtND1), encoded by mitochondrial DNA in P1‐NMCMs infected with Ad‐NC and Ad‐*Sirt4* for 48 h (*n* = 6 biological replicates). Ad‐NC, adenovirus (Ad) harbouring a vector control; Ad‐*Sirt4*, adenovirus (Ad) harbouring *Sirt4*. (C) Immunofluorescence analysis of mitochondria in P1‐NMCMs infected with Ad‐*Sirt4* for 48 h. Mito, mitochondria. Scale bars, 10 μm. (D) Quantification analysis of the number of individual mitochondria in P1‐NMCMs infected with Ad‐NC and Ad‐*Sirt4* for 48 h (*n* = 15 biological replicates for Ad‐NC and *n* = 19 biological replicates for Ad‐*Sirt4*). (E) Quantification analysis of the number of network mitochondria in P1‐NMCMs infected with Ad‐NC and Ad‐*Sirt4* for 48 h (*n* = 18 biological replicates for Ad‐NC and *n* = 19 biological replicates for Ad‐*Sirt4*). (F) Quantification analysis of the number of individual mitochondria compared to network mitochondria in P1‐NMCMs infected with Ad‐NC and Ad‐*Sirt4* for 48 h (*n* = 18 biological replicates for Ad‐NC and *n* = 18 biological replicates for Ad‐*Sirt4*). (G) Transmission electron microscopy images of P1‐NMCMs infected with Ad‐NC and Ad‐*Sirt4* for 48 h. Scale bars, 0.5 μm. M, Mitochondria; S, Sarcomere. (H, I) Quantification analysis of transmission electron microscopy images in G. (J) Flow cytometry analysis of reactive oxygen species (ROS) level in P1‐NMCMs infected with Ad‐*Sirt4* for 48 h (*n* = 4 biological replicates for Ad‐NC and *n* = 5 biological replicates for Ad‐*Sirt4*). (K, L) Immunofluorescence analysis of oxidative DNA damage (indicated by oxidatively modified base 8OHG) and phosphorylated ATM (pATM) expression in P1‐NMCMs infected with Ad‐*Sirt4* for 48 h (*n* = 6 biological replicates). Scale bars, 20 μm. Data are mean ± SEM; *p < 0.05, **p < 0.01, ****p < 0.0001; unpaired two‐tailed t‐tests (B, D–F, H–L).

Sirt4 is reported to localise in mitochondria and our immunofluorescence staining confirmed that Sirt4 was predominantly co‐localised with Mito‐Tracker (a mitochondria marker) (Figure 3A). Mitochondria are strongly associated with oxidative DNA damage which can induce cell cycle arrest [20, 40]. This prompted us to investigate whether Sirt4 overexpression might inhibit cardiomyocyte proliferation and heart regeneration through impairing mitochondria. Therefore, we quantified mitochondrial DNA (mtDNA) content using qRT‐PCR and found a significant reduction in the mtDNA copy number in *Sirt4*‐overexpressed cardiomyocytes (Figure 3B). Mitochondrial morphology analysis using Mito‐Tracker Red staining revealed a significant increase in the proportion of individual mitochondria following Sirt4 overexpression (Figure 3C–F), with no differences in mean network size, mean branch length, median branch length, and mitochondrial footprint in ad‐*Sirt4* infected cardiomyocytes (Figure S9). Subsequently, we examined the mitochondrial microstructure using transmission electron microscopy (TEM) and found that Sirt4 overexpression led to the accumulation of smaller mitochondria with disrupted cristae (Figure 3G–I). These findings demonstrated that Sirt4 overexpression disrupts mitochondrial network integrity.

Multiple studies have established that mitochondrial damage also serves as the primary source of elevated ROS [41, 42]. Flow cytometry analysis using DHE revealed that Sirt4 overexpression induced a significant increase in ROS levels in cardiomyocytes (Figure 3J). Immunofluorescence staining with 8‐hydroxyguanosine (8OHG) and phosphorylated ATM (p‐ATM) demonstrated an increase in oxidative DNA damage and activation of the DNA damage response in cardiomyocytes after Sirt4 overexpression (Figure 3L). These findings indicate Sirt4 disrupts mitochondrial structure, subsequently activating oxidative stress pathways that inhibit cardiomyocyte proliferation.

### 3.4 Sirt4 Deficiency Enhances Juvenile Heart Regeneration

**FIGURE 4:**
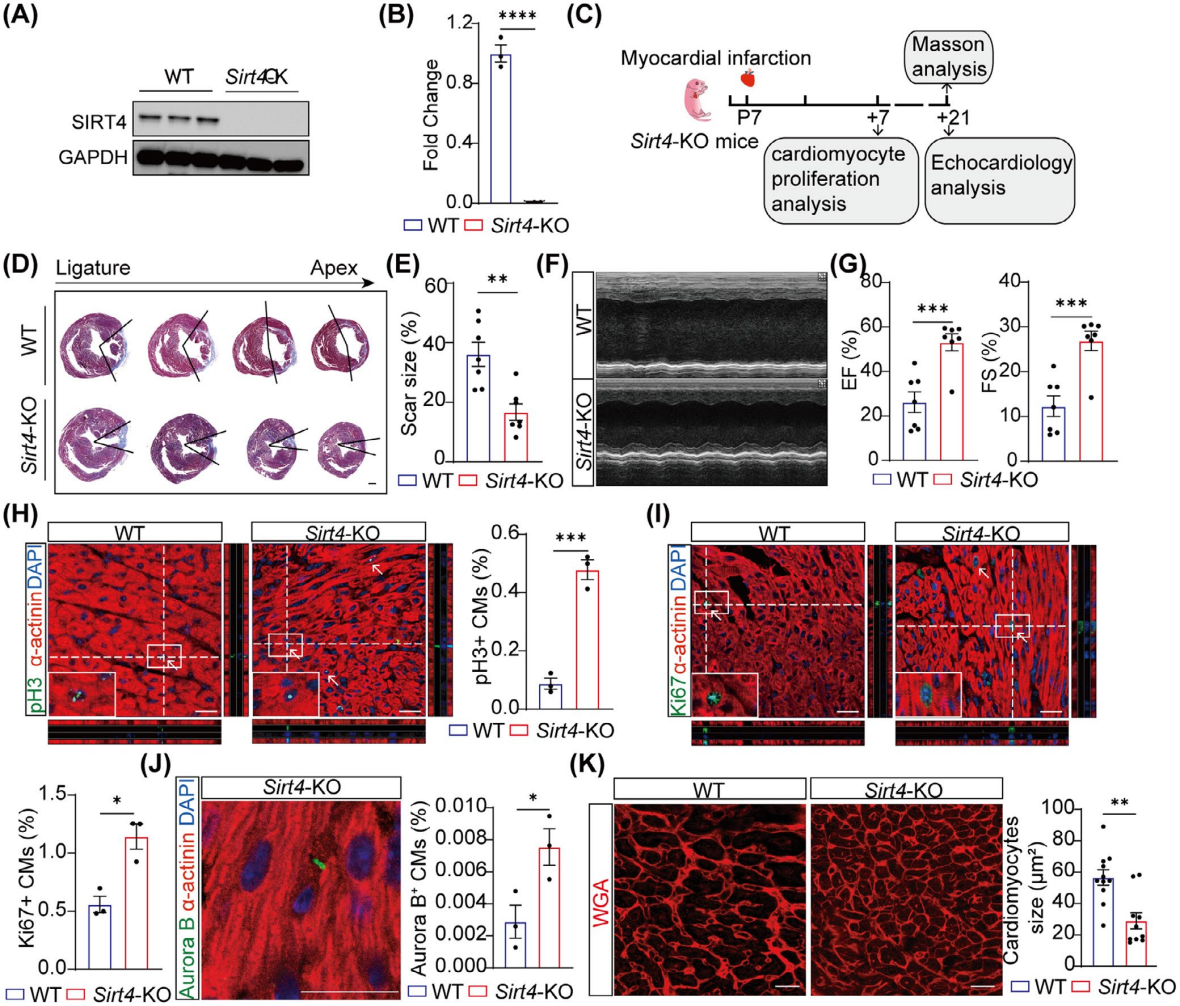
Sirt4 deficiency induces juvenile heart regeneration. (A, B) Western blot analysis (A) and quantification (B) of SIRT4 expression in P7 WT and *Sirt4*‐KO mice (*n* = 3 biological replicates). WT, wild type; *Sirt4*‐KO, *Sirt4* knockout. (C) Schematic representation of the experiment design. Myocardial infarction (MI) was performed on postnatal Day 7 (P7) mice. Hearts were harvested for gene expression and cardiomyocytes (CMs) proliferation analysis at 7‐day post infarction (dpi); and for myocardial fibrosis and echocardiology analysis at 21 dpr. (D, E) Representative Masson trichrome staining of heart cross‐sections and the quantification of fibrosis area proportion of WT and *Sirt4*‐KO mice at 21 dpi (*n* = 7 mice). Scale bars, 400 μm. (F) Representative images of M‐Mode echocardiographic assessment. (G) The ejection fraction (EF) and fractional shortening (FS) of the left ventricle in WT and *Sirt4*‐KO mice at 21 dpi (*n* = 7 mice). (H–J) Immunofluorescence analysis of CM proliferation in heart cross‐sections at 7 dpi (*n* = 3 mice). White arrows indicate pH 3^+^ or Ki67^+^, Aurora B^+^ CMs. Scale bars, 20 μm. (K) Immunofluorescence analysis of CM size in heart cross‐sections at 7 dpi (*n* = 3 mice). Scale bars, 20 μm. Data are mean ± SEM; *p < 0.05, **p < 0.01, ***p < 0.001, ****p < 0.0001; unpaired two‐tailed *t*‐tests (B, E, G–K).

To uncover whether Sirt4 deficiency promoted heart regeneration, we introduced Sirt4 global knockout mice (*Sirt4*‐KO) and confirmed their genetic backgrounds by PCR‐based genotyping (Figure S10A). qRT‐PCR and western blot showed that Sirt4 was effectively knocked out in *Sirt4*‐KO mice heart compared to wild type (WT) mice (Figure 4A,B, Figure S10B,C). Haematoxylin–Eosin (H&E) staining showed no significant morphological differences between *Sirt4*‐KO mice and WT mice at P7 (Figure S11A). We performed MI in *Sirt4*‐KO and WT mice at P7 (Figure 4C), a time point associated with the absence of cardiac regenerative ability [43]. Sham‐operated mice were subjected to the same procedures without LAD ligation. An increased trend was found in the survival rate of *Sirt4*‐KO mice compared to WT mice after MI (Figure S11B). Masson's trichrome staining revealed no significant difference in cardiac phenotypes between WT and *Sirt4*‐KO mice in the sham‐operated group (Figure S11C). However, there was a significantly reduced fibrotic area with increased end‐diastolic thickness at border and remote regions of LV in *Sirt4*‐KO hearts at 21 days post infarction (dpi) compared to WT mice (Figure 4D,E, Figure S11D–F).

Echocardiographic analysis revealed no significant differences in cardiac function between sham‐operated WT and *Sirt4*‐KO mice, as demonstrated by comparable EF and FS values (Figure S11G). However, *Sirt4*‐KO mice exhibited significantly improved cardiac function at 21 dpi, as demonstrated by increased EF and FS values and decreased E/e’ ratio (Figure 4F,G, Figure S11H). Both LVESD and LVEDD were reduced in *Sirt4*‐KO mice compared to WT mice (Figure S11I,J). Meanwhile, LVPW was increased in *Sirt4*‐KO mice compared to WT mice (Figure S11K). Heart rate remained unchanged in *Sirt4*‐KO mice at 21 dpi (Figure S11L). These results suggested that Sirt4 deficiency reduced myocardial fibrosis and enhanced cardiac functional recovery post‐infarction.

To determine whether cardiomyocyte proliferation was regulated by Sirt4 deficiency, we performed co‐immunostaining for α‐actinin with pH 3, Ki67 and Aurora B. We found an increased tendency for cardiomyocyte proliferation (pH 3^+^ cardiomyocytes) of *Sirt4*‐KO mice at 7‐day post sham operation (Figure S11M). However, pH 3‐, Ki67‐ and Aurora B‐positive cardiomyocytes were significantly increased in *Sirt4*‐KO mice at 7 dpi relative to WT mice (Figure 4H–J). WGA staining revealed a significant decrease in cardiomyocyte cell size in *Sirt4*‐KO mice (Figure 4K). These findings conclusively demonstrate that loss of Sirt4 promotes cardiomyocyte proliferation and extends the time window of heart regeneration.

### 3.5 Sirt4 Deficiency Promotes Adult Cardiac Repair

**FIGURE 5:**
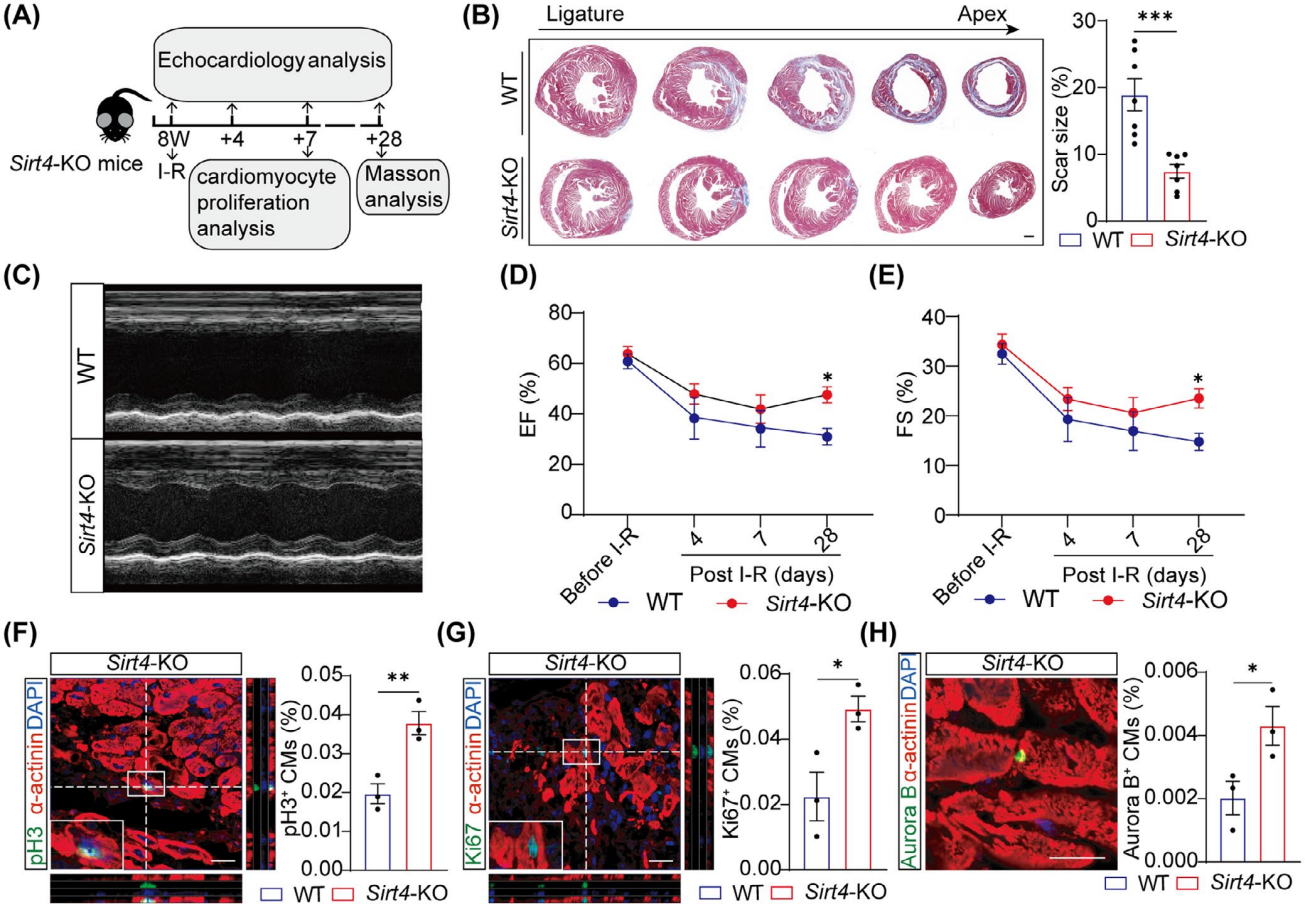
Sirt4 deficiency promotes heart regeneration in adult mice. (A) Schematic representation of the experiment design. Ischemia–reperfusion (I‐R) was performed in 8 weeks mice. The hearts were harvested for cardiomyocyte (CM) proliferation analysis at 7‐day post I‐R; for scar size analysis with Masson trichrome staining at 28‐day post I‐R. Echocardiology analysis was performed at before and 4, 7 and 28 days post I‐R. WT, wild type; *Sirt4*‐KO, *Sirt4* knockout. (B) Representative Masson's trichrome staining of heart cross‐sections and the quantification of fibrosis area proportion of WT and *Sirt4*‐KO mice at 28‐day post I‐R (*n* = 7 mice). Scale bars, 500 μm. WT, wild type. (C) Representative images of M‐Mode echocardiographic assessment. (D, E) Ejection fraction (EF) and fractional shortening (FS) of the left ventricle in WT and *Sirt4*‐KO mice before and 4, 7 and 28 days post I‐R (*n* = 7 mice). (F–H) Immunofluorescence analysis of CM proliferation in heart cross‐sections at 7‐day post I‐R (*n* = 3 mice). White arrows indicate pH 3^+^ or Ki67^+^, Aurora B^+^ CMs. Scale bars, 20 μm. Data are mean ± SEM; *p < 0.05, **p < 0.01, ***p < 0.001; unpaired two‐tailed *t*‐tests (B, F–H); two‐way ANOVA (D, E).

To further investigate whether Sirt4 could be a potential target for heart repair in adult mice, we performed ischemia–reperfusion (I‐R) in adult *Sirt4*‐KO mice and analysed the effects of *Sirt4* KO on cardiomyocyte proliferation, myocardial fibrosis and cardiac function (Figure 5A). H&E staining showed no significant morphological differences between *Sirt4*‐KO and WT mice at P56 (Figure S12A). We found that Sirt4 deficiency did not alter survival rate post‐I‐R (Figure S12B). Masson's trichrome staining showed no significant difference in cardiac phenotypes between adult WT and *Sirt4*‐KO mice in the sham‐operated group (Figure S12C), while *Sirt4* KO significantly reduced the infarct size at 28days post I‐R (Figure 5B, Figure S12D), with no significant differences in LV end‐diastolic thickness (Figure S12E,F).

Comparable cardiac function was found between WT and *Sirt4*‐KO mice in the sham‐operated group, as evidenced by similar EF and FS values (Figure S12G). However, serial echocardiographic assessment revealed that *Sirt4‐KO* improved cardiac function, as EF and FS values were significantly higher than those in the WT mice, with comparable heart rate at 28days post I‐R (Figure 5C–E, Figure S12H). Both LVESD and LVEDD were reduced in *Sirt4*‐KO mice compared to WT mice (Figure S12I,J). Meanwhile, LVPW showed no significant differences in *Sirt4*‐KO mice (Figure S12K).

To reveal whether cardiomyocyte proliferation is responsible for the roles of *Sirt4* deficiency during cardiac repair, we performed co‐immunostaining for α‐actinin with pH 3, Ki67 and Aurora B. The results showed an increased tendency for cardiomyocyte proliferation (pH 3^+^ cardiomyocytes) of *Sirt4*‐KO mice at 7days post sham operation (Figure S12L). However, the percentages of pH 3‐, Ki67‐ or Aurora B‐positive cardiomyocytes were significantly increased in *Sirt4*‐KO mice at 7 days post I‐R, indicating that *Sirt4* deletion significantly enhanced cardiomyocyte proliferation following I‐R (Figure 5F–H). These results demonstrate that loss of *Sirt4* induces cardiomyocyte proliferation and promotes heart regeneration after myocardial I‐R injury in adult mice, suggesting the role of Sirt4 as a potential therapeutic target for cardiac injury.

## 4 Discussion

Despite extensive research, the molecular mechanisms underlying the loss of proliferative capacity in cardiomyocytes during early postnatal stages remain poorly understood. Our study identifies Sirt4 as a critical regulator of cardiomyocyte proliferation and heart regeneration. *Sirt4* overexpression induced mitochondrial dysfunction and oxidative DNA damage, thereby inhibiting cardiomyocyte proliferation and impairing neonatal heart regeneration. Conversely, Sirt4 deficiency promoted cardiomyocyte proliferation and improved cardiac repair in both juvenile and adult injury models. These findings suggest the therapeutic potential for Sirt4 inhibition in heart regeneration.

Sirt4 is highly expressed in the heart, kidney, liver, and brain, implicating its possible roles in these tissues [20, 44]. Emerging evidence indicates that Sirt4 loss‐of‐function promotes glutamine‐dependent proliferation and induces genomic instability under stress conditions, potentially contributing to tumorigenesis [45]. In the cardiovascular system, Sirt4 deficiency has been shown to confer protection against angiotensin II‐induced cardiac hypertrophy and fibrosis [20]. Furthermore, recent studies reveal that Sirt4 participates in ammonia detoxification by regulating amino acid catabolism [45], underscoring its multifaceted roles in metabolic regulation. Our current study significantly expands the functional repertoire of Sirt4 by demonstrating its critical involvement in cardiomyocyte proliferation and cardiac regeneration. We established that Sirt4 overexpression potently suppressed cardiomyocyte proliferation both in vitro and in vivo, ultimately impairing the neonatal heart's regenerative capacity following injury. These findings position Sirt4 as a key regulator that integrates mitochondrial function with cellular proliferation dynamics in the heart.

Several regulators of postnatal cardiomyocyte cell cycle arrest have been described thus far, including Meis1–Hoxb13 [26], succinate [46], CBX7 [47] and so on. Meis1–Hoxb13 double‐knockout leads to widespread cardiomyocyte proliferation and improved left ventricular systolic function in adult mice following MI [26]. The inhibition of succinate dehydrogenase by malonate results in a robust regenerative response in the adult mouse heart after MI [46]. Our work adds Sirt4 to this negative regulator list, with translational implications. The improved regeneration in *Sirt4*‐KO adult hearts is particularly promising for therapeutic development.

Sirt4's mitochondrial localization positions it to regulate ROS production and DDR activation. ROS impacts nearly all key aspects of cardiac maladaptation, including heart failure [48, 49, 50], contractile dysfunction [51, 52], extracellular matrix remodelling [53, 54] and arrhythmia [52, 55]. The oxygen‐rich postnatal environment induces cardiomyocyte cell‐cycle arrest through DNA damage response [8], which has emerged as an important factor in the loss of heart regenerative response. Sirt4 has been reported to promote hypertrophic growth, fibrosis generation, and cardiac dysfunction by increasing ROS levels under pathological conditions [20]. In this study, we demonstrated a critical role of Sirt4 in mitochondrial abnormalities and ROS accumulation in the myocardium, which induced the activation of DNA damage response, thereby inhibiting cardiomyocyte proliferation and heart regeneration. Additionally, a recent study shows that Sirt4 could also translocate into nucleus, which mediates deacetylation of U2AF2 to modulate renal fibrosis [56]. Therefore, the functions of Sirt4 beyond the mitochondrial location also can be considered in further study.

In conclusion, we identify Sirt4 as a novel regulator of cardiomyocyte proliferation and cardiac repair. *Sirt4*‐Tg mice exhibit rapid onset of cardiac dysfunction and fibrosis post myocardial injury. However, Sirt4 deficiency improves cardiac function and reduces myocardial fibrosis through cardiomyocyte proliferation, showing a remarkable pro‐repair response. The present study provides novel mechanistic insights into the likely link between Sirt4 and heart regeneration, offering new therapeutic perspectives for ischaemic heart diseases.

## Author Contributions

**Weijing Liu:** conceptualization (equal), data curation (equal), formal analysis (equal), methodology (equal), project administration (equal), software (equal), validation (equal), visualization (equal), writing – original draft (equal). **Jie Feng:** data curation (equal), formal analysis (equal), project administration (equal), resources (equal), software (equal), writing – original draft (equal). **Yuan Zhang:** data curation (equal), methodology (equal), resources (equal), software (equal), visualization (equal), writing – original draft (equal). **Yanyan Hao:** methodology (equal), supervision (equal). **Jiajun Zhong:** investigation (equal), validation (equal). **Xinchang Liu:** software (equal), validation (equal). **Dongcheng Cai:** investigation (equal), software (equal). **Haorui Liu:** data curation (equal), resources (equal). **Lina Bai:** funding acquisition (equal), visualization (equal). **Miaoqing Hu:** data curation (equal), resources (equal). **Hong Lian:** data curation (equal), resources (equal). **Yu Nie:** conceptualization (equal), funding acquisition (equal), writing – review and editing (equal). **Houzao Chen:** conceptualization (equal), project administration (equal), writing – review and editing (equal). **Yuyao Wang:** conceptualization (equal), funding acquisition (equal), project administration (equal), writing – review and editing (equal).

## Conflicts of Interest

The authors declare no conflicts of interest.

## Supporting information


Data S1.

## Data Availability

The data sets used and analysed during the current study are available from the corresponding author on reasonable request. The RNA‐seq dataset generated in this study has been deposited in the National Center of Biotechnology Information with accession number PRJNA1159548 available.
