# Supplementary material for: Sirt4 Deficiency Promotes Cardiomyocyte Proliferation and Cardiac Repair
Source: J Cell Mol Med. 2025 Aug 21;29(16):e70741. doi: 10.1111/jcmm.70741 (PMC12370541; doi:10.1111/jcmm.70741)
Supplement: Supplementary file 1 — Data S1. [file JCMM-29-e70741-s001.docx]

Supplementary Information for

**Sirt4 deficiency promotes cardiomyocyte proliferation and cardiac repair**

Weijing Liu, Jie Feng, Yuan Zhang, Yanyan Hao, Jiajun Zhong, Xinchang Liu, Dongcheng Cai, Haorui Liu, Lina Bai, Miaoqing Hu, Hong Lian, Yu Nie, Houzao Chen, Yuyao Wang

**This file includes:**

Figure S1-S12

Table S1-S4


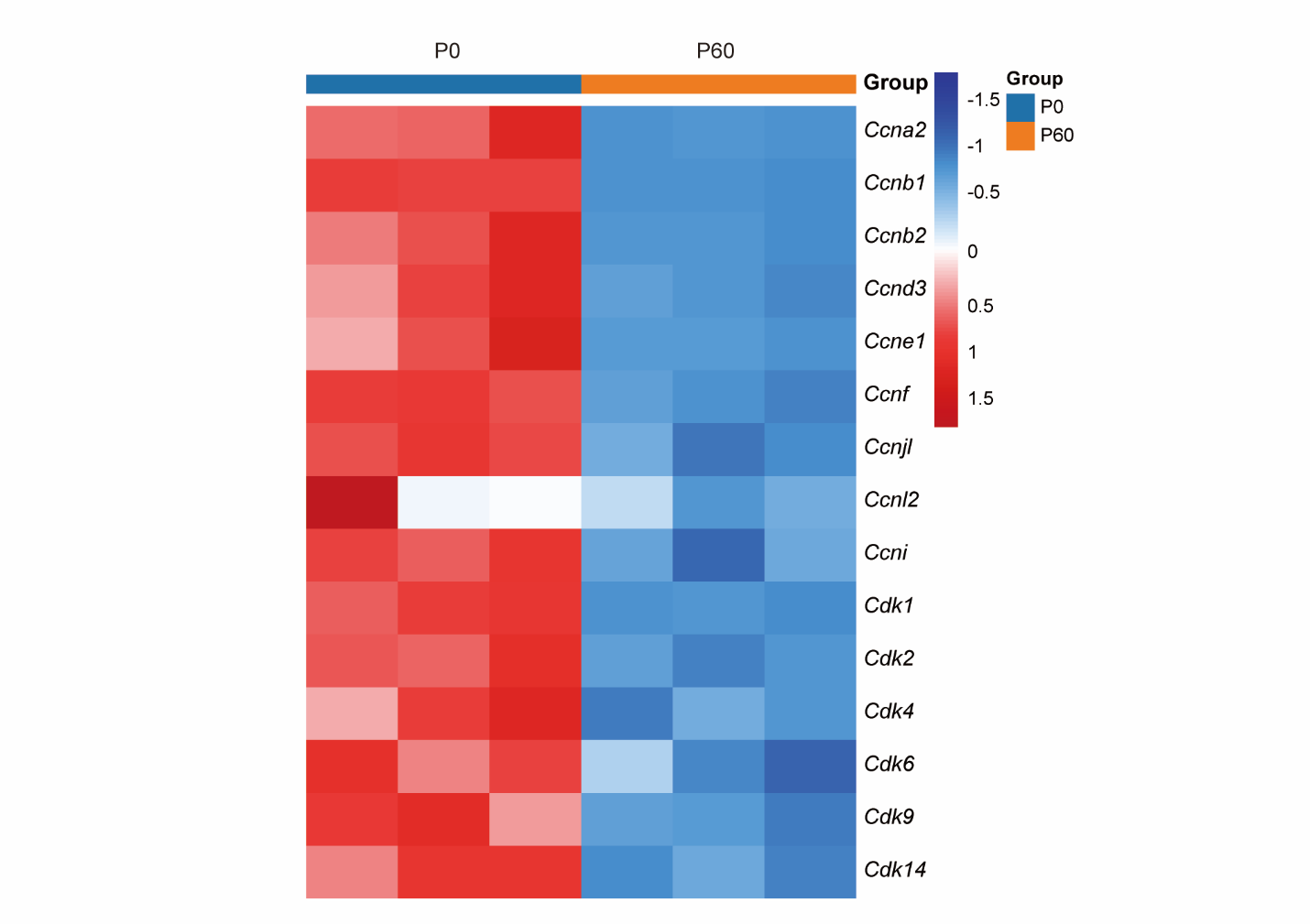


**FIGURE S1 The expression of cell cycle genes during cardiac development.** Heatmap showing RNA expression of cell cycle genes in mouse heart samples during cardiac development based on RNA sequencing data (n = 3 biological replicates). P0, postnatal day 0; P60, postnatal day 60; Ccna2, cyclin A2; Ccnb1, cyclin B1; Ccnb2, cyclin B2; Ccnd3, cyclin D3; Ccne1, cyclin E1; Ccnf, cyclin F; Ccnjl, cyclin J-like; Ccnl2, cyclin L2; Ccni, cyclin I; Cdk1, cyclin dependent kinase 1; Cdk2, cyclin dependent kinase 2; Cdk4, cyclin dependent kinase 4; Cdk6, cyclin dependent kinase 6; Cdk9, cyclin dependent kinase 9; Cdk14, cyclin dependent kinase 14.


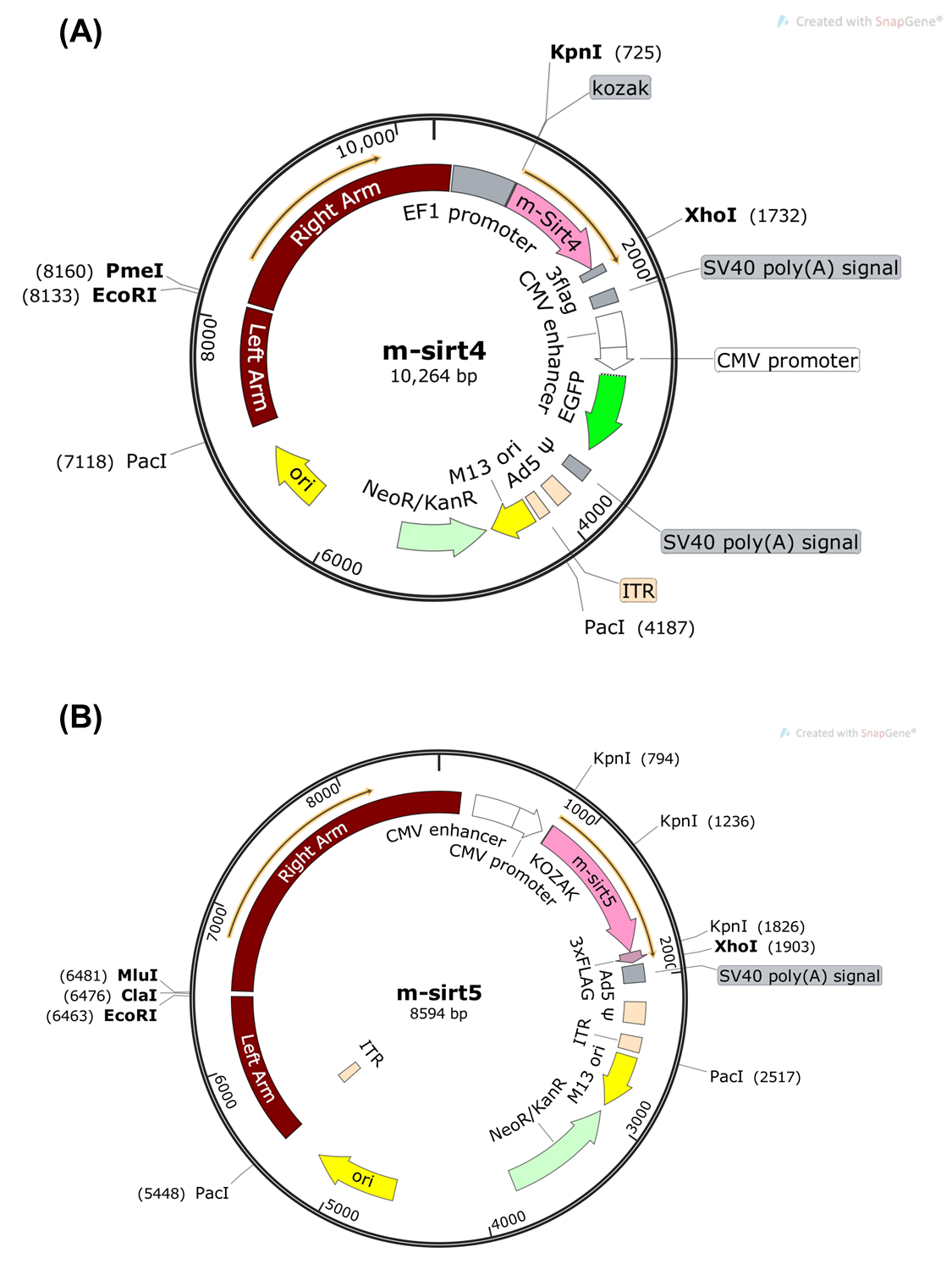


**FIGURE S2 Plasmid construction profile of *Sirt4* and *Sirt5* overexpressing adenovirus.**


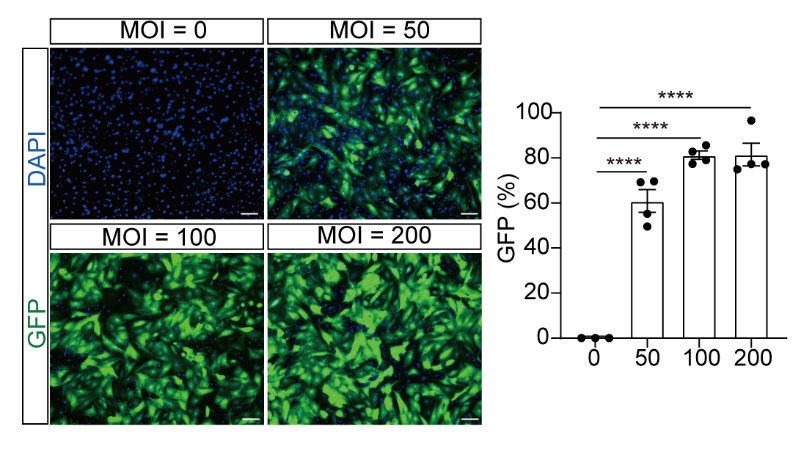


**FIGURE S3 Transfection efficiency of adenoviruses with different MOI.** We isolated P1 neonatal mouse cardiomyocytes (P1-NMCMs) and treated them with adenovirus for *Sirt4* overexpression at three different multiplicities of infection (MOI) for 48 hours (MOI: 50, 100, and 200), followed by quantitative assessment of transduction efficiency through GFP-positive cell counting. Scare bar, 100 μm. Data are mean ± SEM; *****p* < 0.0001; Multiple comparisons were made using one-way ANOVA.


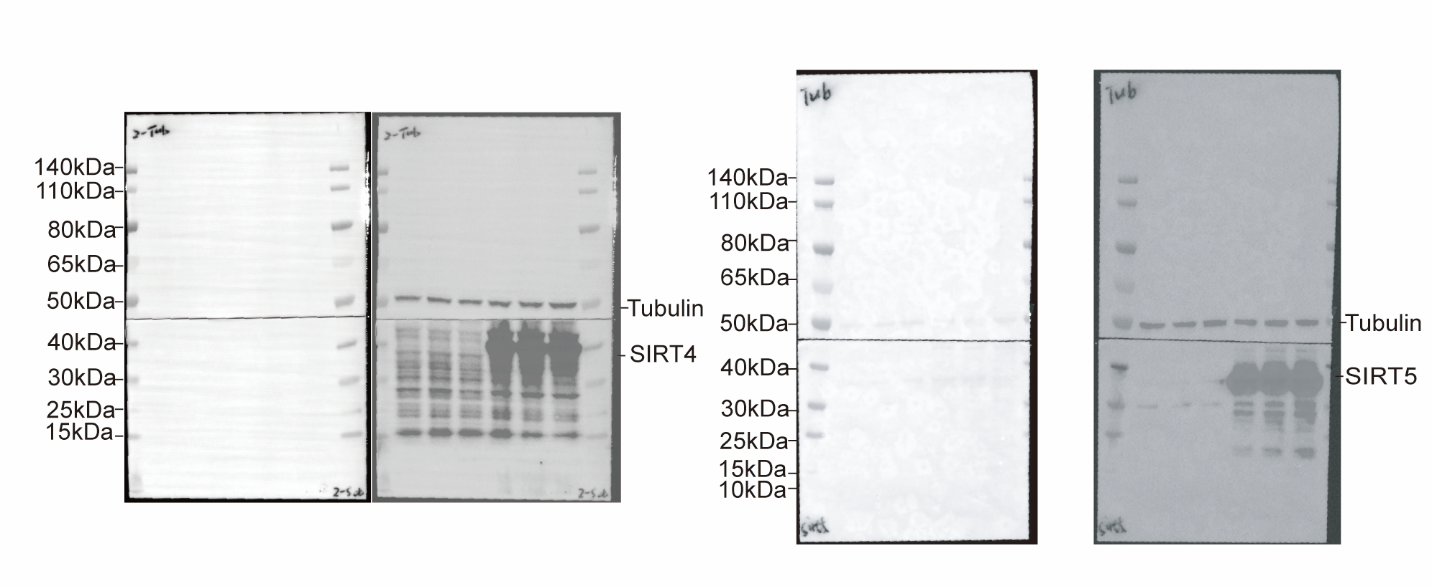


**FIGURE S4 Images of unprocessed blots used for Figure 1F.**


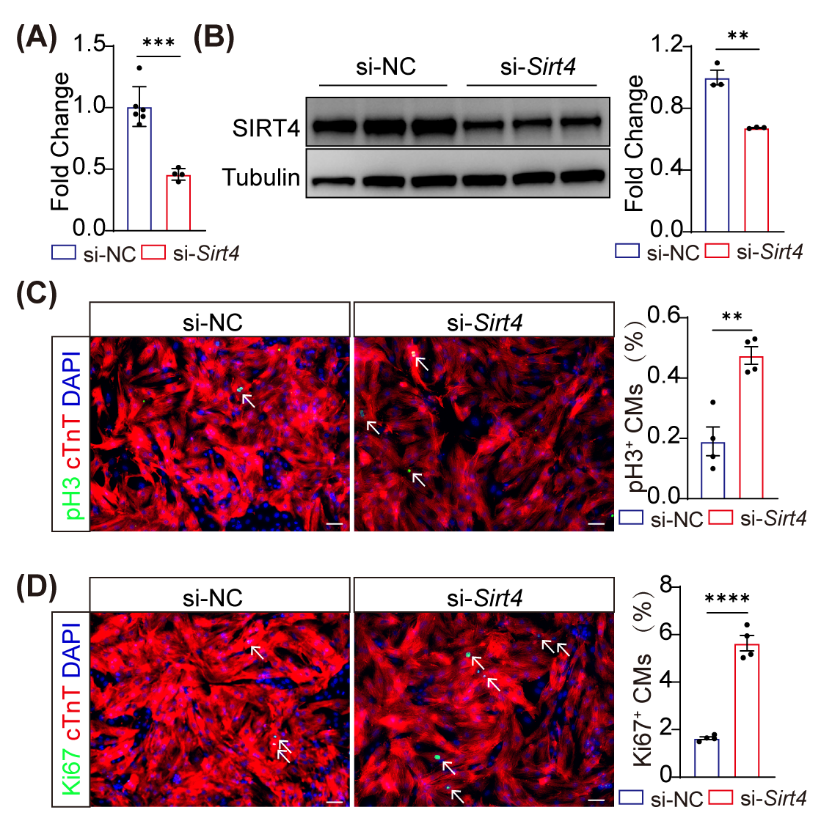


**FIGURE S5 *Sirt4* knockdown promotes cardiomyocyte proliferation *in vitro.*** (A) Quantitative real-time polymerase chain reaction (qRT-PCR) analysis of *Sirt4* expression in P1 neonatal mice cardiomyocytes (P1-NMCMs) transfected with si-NC and si-*Sirt4* for 48 hours (n = 6 biological replicates for si-NC and n = 4 biological replicates for si-*Sirt4*). (B) Western blot analysis of SIRT4 expression in P1-NMCMs transfected with si-NC and si-*Sirt4* for 48 hours (n = 3 biological replicates). (C, D) Immunofluorescence analysis of the proliferation of P1-NMCMs transfected with si-NC and si-*Sirt4* for 48 hours (n = 4 biological replicates). White arrows indicate pH3^+^, Ki67^+^ CMs. Scale bars, 20 μm. Data are mean ± SEM; ***p* < 0.01, ****p* < 0.001, *****p* < 0.0001; unpaired two-tailed *t*-tests (A-D).


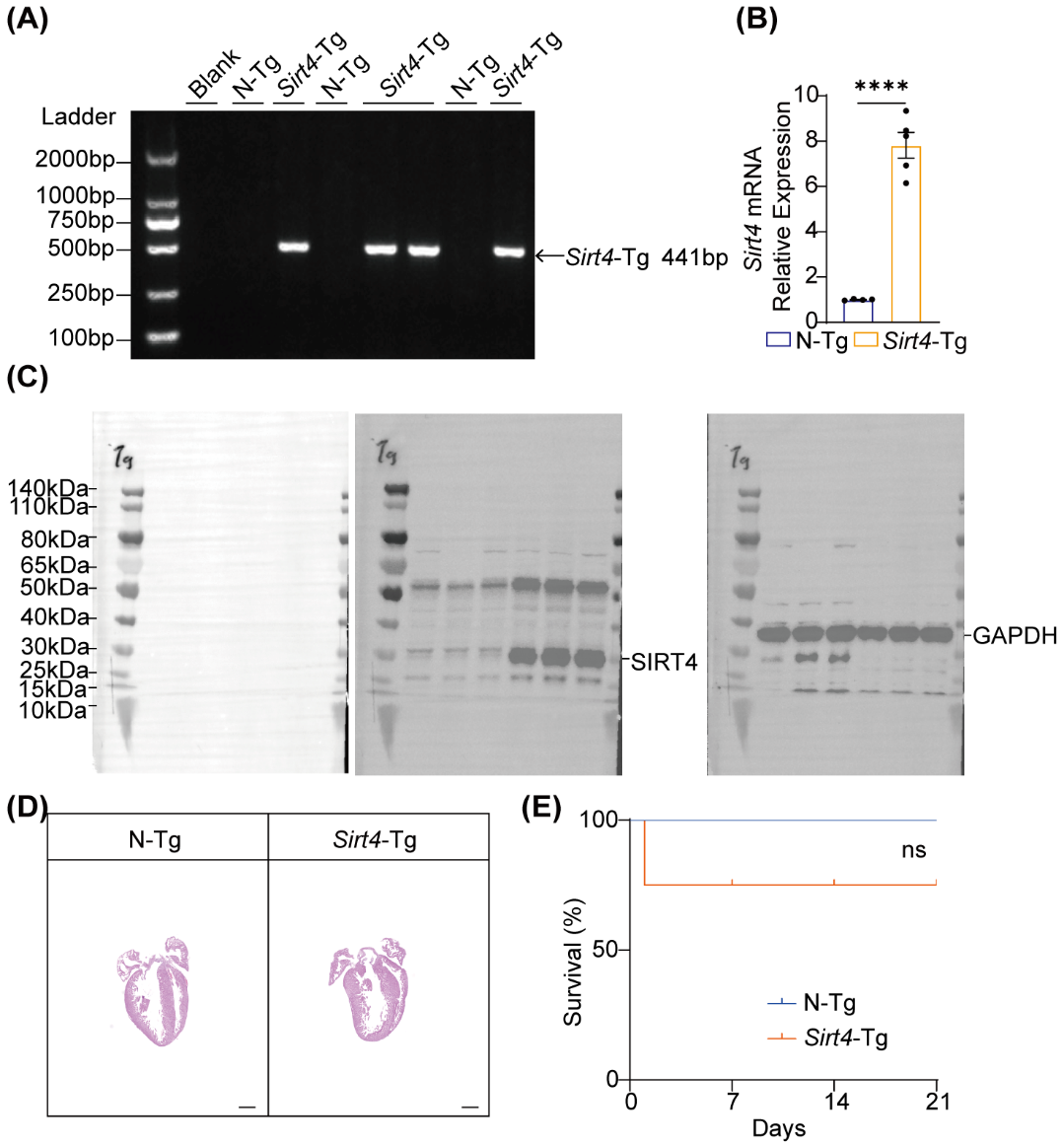


**FIGURE S6 The expression of Sirt4 and cardiac morphology in *Sirt4*-Tg mice.** (A) Genotyping of *Sirt4*-Tg mice and the littermates. (B) Quantitative real-time polymerase chain reaction (qRT-PCR) analysis of Sirt4 expression in N-Tg and *Sirt4*-Tg mice at 7-day post resection (n = 5 biological replicates). N-Tg, negative transgenic; *Sirt4*-Tg, *Sirt4* transgenic. (C) Images of unprocessed blots used for Figure 2B. (D) HE staining images of physiological status of *Sirt4*-Tg and N-Tg mice heart at P1. (E) Survival rate of *Sirt4*-KO and WT mice at 21dpr (n = 9 mice). Scale bars, 500 μm. Data are mean ± SEM; ns, not significance; *****p* < 0.0001; unpaired two-tailed *t*-tests (B).


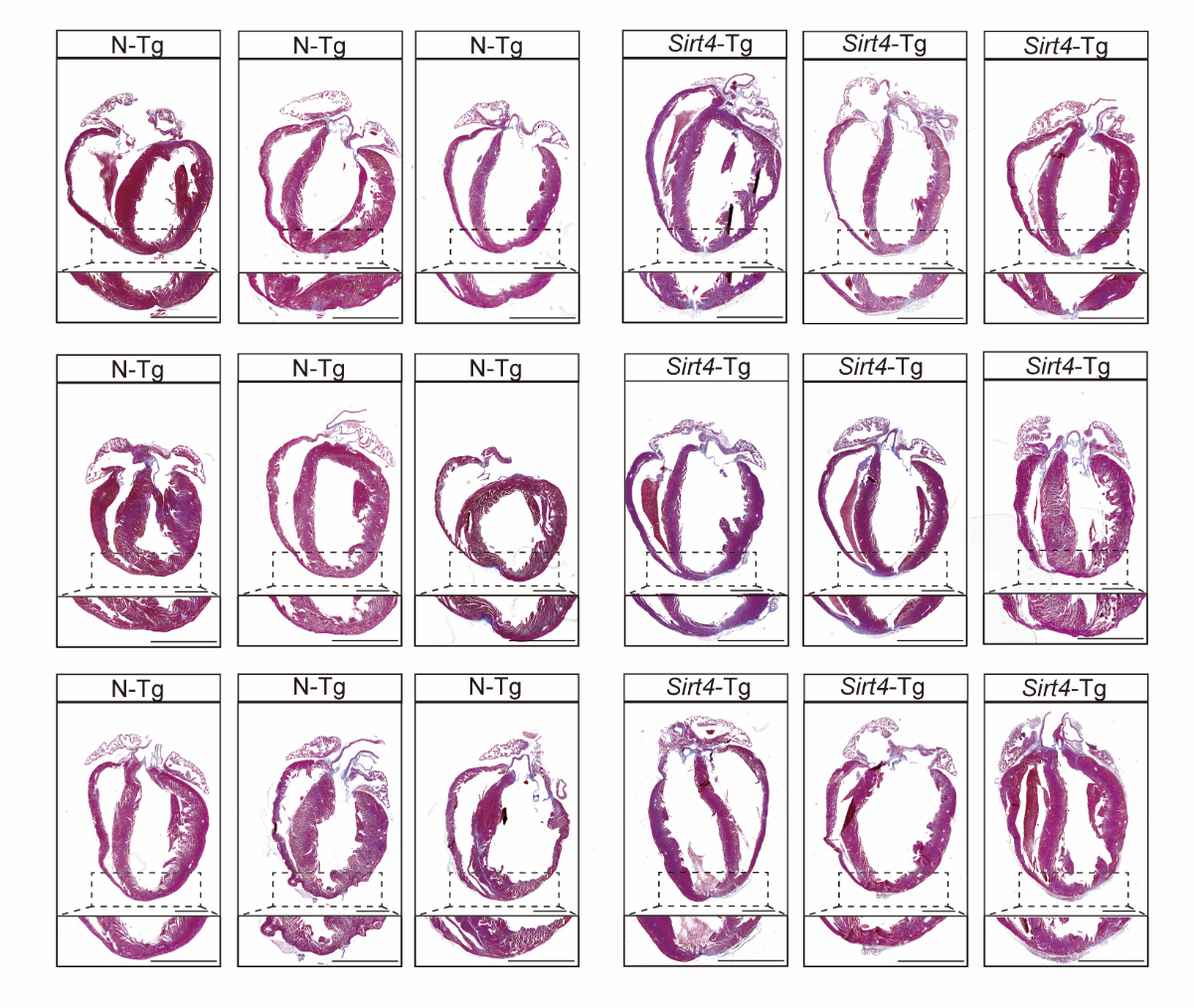


**FIGURE S7 Images of Masson trichrome staining used for Figure 2D.** Masson trichrome staining of heart sections of N-Tg and *Sirt4*-Tg mice at 21-day post resection (n = 9 mice). N-Tg, negative transgenic; *Sirt4*-Tg, *Sirt4* transgenic. Scale bars, 400 μm.


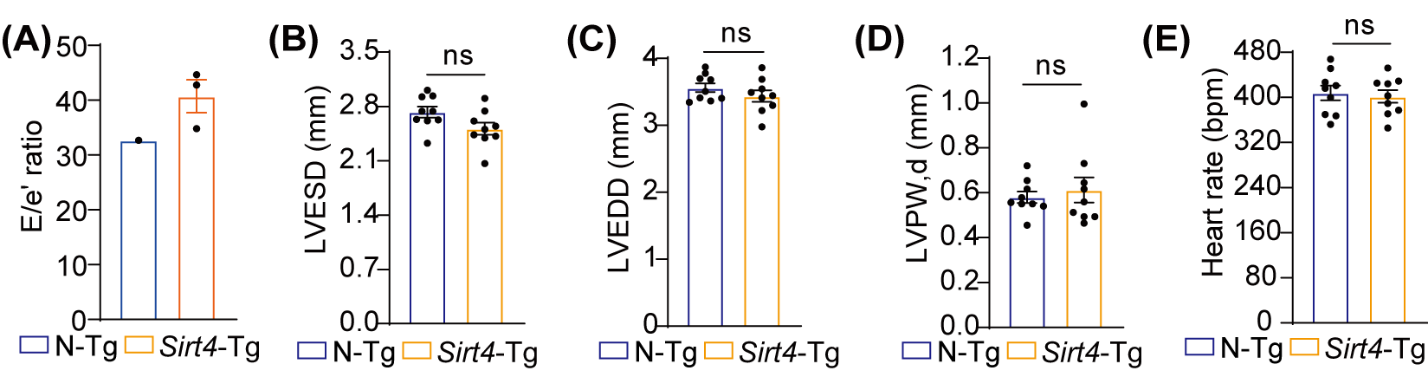


**FIGURE S8 Cardiac function in *Sirt4*-Tg mice at 21 dpr.** (A) The diastolic function (E/e’ ratio) in N-Tg and *Sirt4*-Tg mice at 21 dpr (n = 1 mice for N-Tg mice and n = 3 for *Sirt4*-Tg mice). (B) The LV end-systolic diameter (LVESD) in N-Tg and *Sirt4*-Tg mice at 21 dpr (n = 9 mice). (C) The LV end-diastolic diameter (LVEDD) in N-Tg and *Sirt4*-Tg mice at 21 dpr (n = 9 mice). (D) The LV posterior wall thickness at end-diastole (LVPW) in N-Tg and *Sirt4*-Tg mice at 21 dpr (n = 9 mice). (E) The heart rate in N-Tg and *Sirt4*-Tg mice at 21 dpr (n = 9 mice). Data are mean ± SEM; ns, not significance; unpaired two-tailed *t*-tests (B-E).


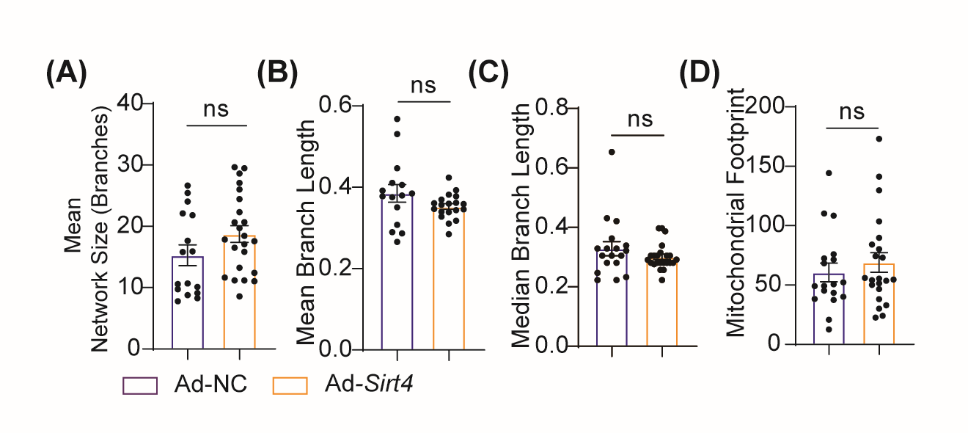


**FIGURE S9 The quantification analysis of mitochondrial networks**. (A) Quantification analysis of mean networks size in P1-NMCMs infected with Ad-NC and Ad-*Sirt4* for 48 hours (n = 16 biological replicates for Ad-NC and n = 23 biological replicates for Ad-*Sirt4*). Ad-*Sirt4*, adenovirus (Ad) harboring *Sirt4*. (B, C) Quantification analysis of mean and median branch length in P1-NMCMs infected with Ad-NC and Ad-*Sirt4* for 48 hours (n = 16 biological replicates for Ad-NC and n = 19 biological replicates for Ad-*Sirt4*). (D) Quantification analysis of mitochondrial footprint in P1-NMCMs infected with Ad-NC and Ad-*Sirt4* for 48 hours (n = 18 biological replicates for Ad-NC and n = 22 biological replicates for Ad-*Sirt4*). Data are mean ± SEM; ns, not significance; unpaired two-tailed *t*-tests (A-D).


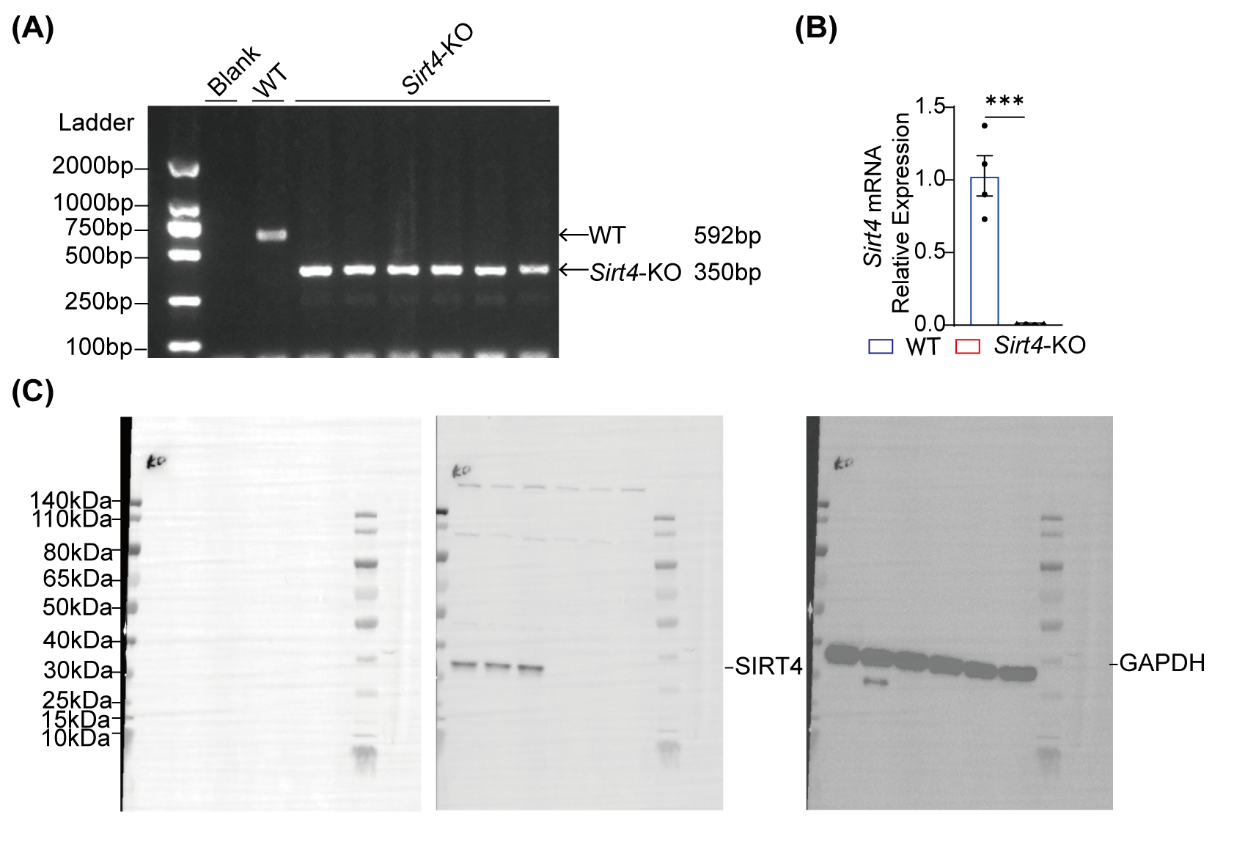


**FIGURE S10 The expression of Sirt4 in *Sirt4*-KO mice.** (A) Genotyping of *Sirt4*-KO mice and the littermates. (B) Quantitative real-time polymerase chain reaction (qRT-PCR) analysis of Sirt4 expression in WT and *Sirt4*-KO mice at postnatal day 7 (n = 4 biological replicates). WT, wild type; *Sirt4*-KO, *Sirt4* knockout. (C) Images of unprocessed blots used for Figure 4B. Data are mean ± SEM; ****p* < 0.001; unpaired two-tailed *t*-tests (B).


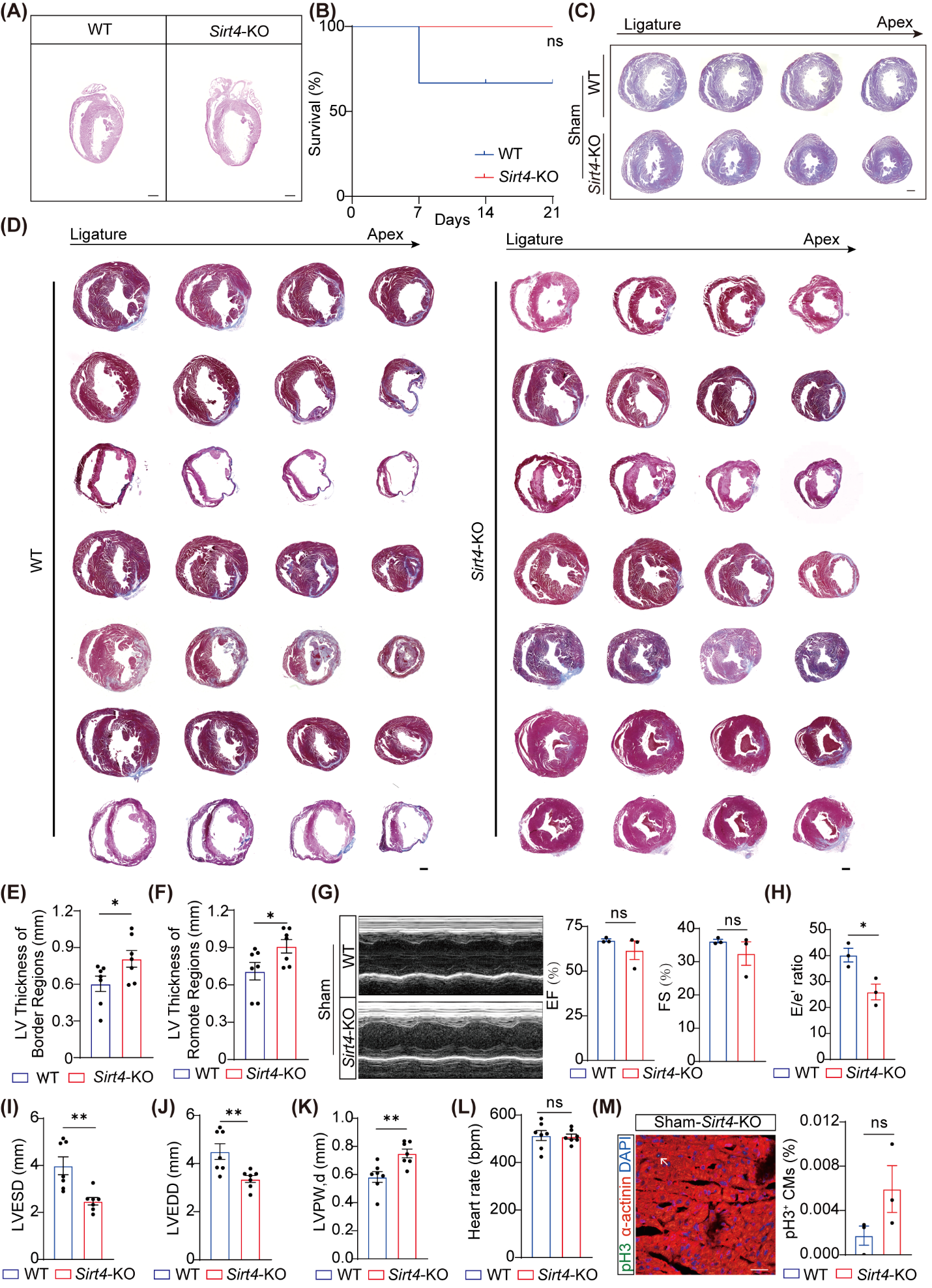


**FIGURE S11 Sirt4 deficiency enhances juvenile heart regeneration.** (A) H&E staining images of physiological status of *Sirt4*-KO and WT mice heart at P7. Scale bars, 500 μm. WT, wild type; *Sirt4*-KO, *Sirt4* knockout. (B) Survival rate of *Sirt4*-KO and WT mice at 21dpi (n = 7 mice). (C) Representative Masson trichrome staining of heart cross-sections of WT and *Sirt4*-KO mice at 21 dps (n = 3 mice). Scale bars, 500 μm. (D) Masson trichrome staining of heart cross-sections of WT and *Sirt4*-KO mice at 21-day post infarction performed at postnatal day 7 (n = 7 mice). Scale bars, 400 μm. (E) The LV thickness of border regions in WT and *Sirt4*-KO mice at 21 dpi (n = 7 mice). (F) The LV thickness of romote regions in WT and *Sirt4*-KO mice at 21 dpi (n = 7 mice). (G) Representative images of M-Mode echocardiographic assessment and ejection fraction (EF) and fraction shortening (FS) of the left ventricle in WT and *Sirt4*-KO mice at 21 dps (n = 3 mice). (H) The diastolic function (E/e’ ratio) in WT and *Sirt4*-KO mice at 21 dpi (n = 3 mice). (I) The LV end-systolic diameter (LVESD) in WT and *Sirt4*-KO mice at 21 dpi (n = 7 mice). (J) The LV end-diastolic diameter (LVEDD) in WT and *Sirt4*-KO mice at 21 dpi (n = 7 mice). (K) The LV posterior wall thickness at end-diastole in WT and *Sirt4*-KO mice at 21 dpi (n = 7 mice). (L) The heart rate in WT and *Sirt4*-KO mice at 21 dpi (n = 7 mice). (M) Immunofluorescence analysis of CM proliferation in heart cross-sections at 7 dps (n = 3 mice). White arrows indicate pH3^+^ CMs. Scale bars, 20 μm. Data are mean ± SEM; **p* < 0.05, ***p* < 0.01, ns, not significance; unpaired two-tailed t-tests (E-M).


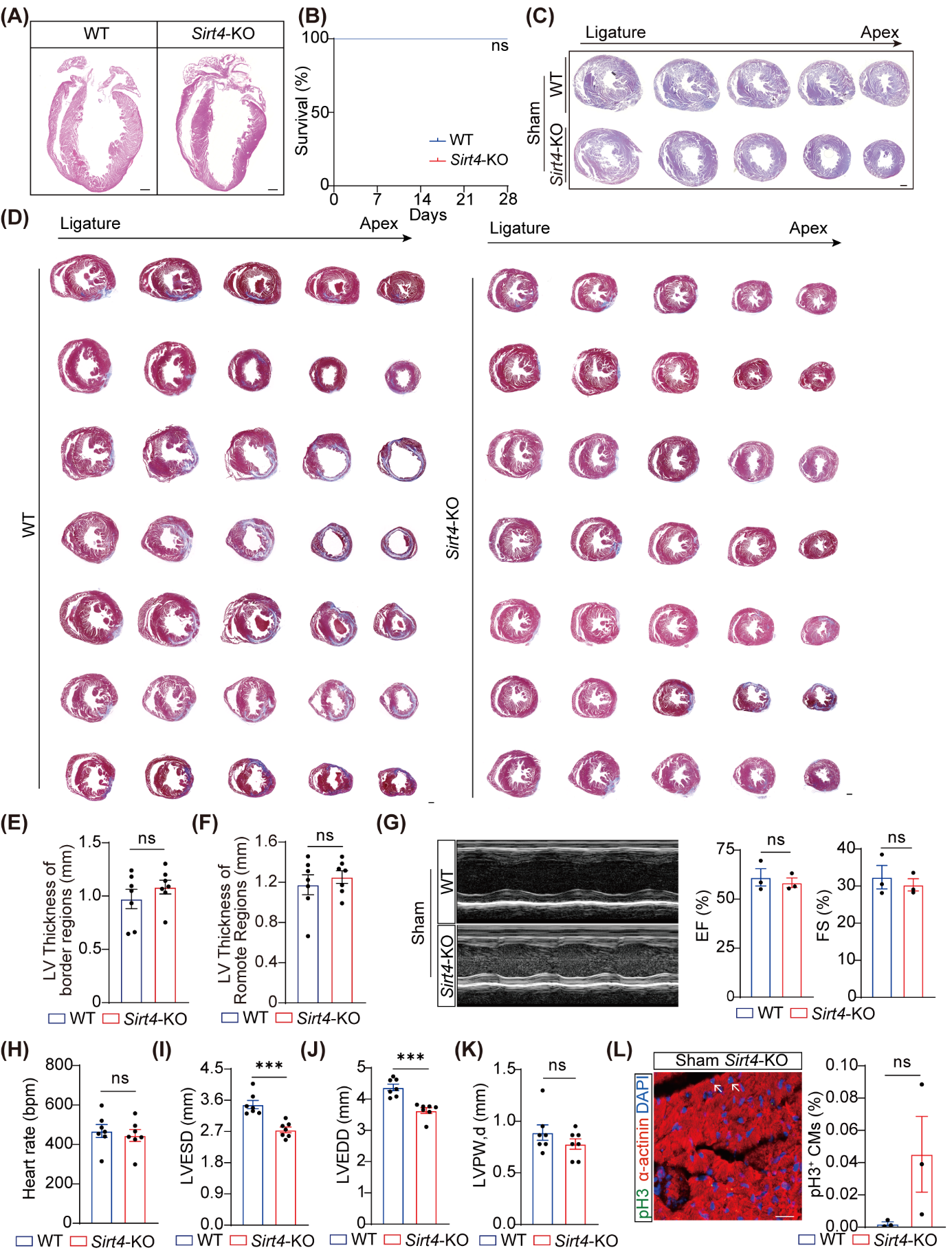


**FIGURE S12 Sirt4 deficiency promotes adult cardiac repair.** (A) H&E staining images of physiological status of *Sirt4*-KO and WT mice heart at P56. Scale bars, 500 μm. (B) Survival rate of *Sirt4*-KO and WT mice at 28 days post I-R (n = 7 mice). (C) Representative Masson trichrome staining of heart cross-sections of WT and *Sirt4*-KO mice at 28 dps (n = 3 mice). Scale bars, 500 μm. (D) Masson trichrome staining of heart cross-sections of WT and *Sirt4*-KO mice at 28-day post I-R performed at 8 weeks old (n = 7 mice). Scale bars, 500 μm. (E) The LV thickness of border regions in WT and *Sirt4*-KO mice at 21 days post I-R (n = 7 mice). (F) The LV thickness of romote regions in WT and *Sirt4*-KO mice at 21 days post I-R (n = 7 mice). (G) Representative images of M-Mode echocardiographic assessment and ejection fraction (EF) and fraction shortening (FS) of the left ventricle in WT and *Sirt4*-KO mice at 28 dps (n = 3 mice). (H) The heart rate in WT and *Sirt4*-KO mice at 28 day-post I-R (n = 7 mice). (I) The LV end-systolic diameter (LVESD) in WT and *Sirt4*-KO mice at 28 day-post I-R (n = 7 mice). (J) The LV end-diastolic diameter (LVEDD) in WT and *Sirt4*-KO mice at 28 day-post I-R (n = 7 mice). (K) The LV posterior wall thickness at end-diastole in WT and *Sirt4*-KO mice at 28 day-post I-R (n = 7 mice). (L) Immunofluorescence analysis of CM proliferation in heart cross-sections at 7 dps (n = 3 mice). White arrows indicate pH3^+^ CMs. Scale bars, 20 μm. Data are mean ± SEM; ****p* < 0.001, ns, not significance; unpaired two-tailed t-tests (E-L).

Table S1 Body weight and sex between *Sirt4*-Tg mice, *Sirt4*-KO mice and their littermates.

| **Model** | **Genotype** | **Age** | **Weight (g)** | ***p*-value** | **Sex** |
| --- | --- | --- | --- | --- | --- |
| P1 AR | *Sirt4*-Tg | P1 | 1.44±0.07 | 0.6849 | Mixed-sex |
|  | N-Tg | P1 | 1.39±0.06 |  | Mixed-sex |
| P7 MI | *Sirt4*-KO | P7 | 4.67±0.6 | 0.8178 | Mixed-sex |
|  | WT | P7 | 4.79±0.38 |  | Mixed-sex |
| Adult I-R | *Sirt4*-KO | P56 | 23.30±0.16 | 0.2270 | Male |
|  | WT | P56 | 22.79±0.37 |  | Male |

Table S2 The primers sequence for genotyping.

| **Mice** | | **Primer sequence** |
| --- | --- | --- |
| Sirt4-Tg mice | forward | 5’- TGGGAGAAACTCGGAAAGCTG-3’ |
|  | reverse | 5’- CTCCCAGGCAGTGAGGATAAAC-3’ |
| Sirt4-KO mice | P1 | 5’-TAAAGATAGTTGTAAGTCACC-3’ |
|  | P2 | 5’-AGAGCCCAGTGTGCTGGGTTG-3’ |
|  | P3 | 5’-GTCTGTCCTAGCTTCCTCACTG-3’ |

Table S3 The primers sequence for qRT-PCR.

| **Genes** | | **Primer sequence** |
| --- | --- | --- |
| *Sirt4* | forward | 5’- GAGCAACTGGGAGAGACTGG-3’ |
|  | reverse | 5’- ACAGCACGGGACCTGAAA-3’ |
| *Sirt5* | forward | 5’- CGCTGGAGGTTACTGGAGA-3’ |
|  | reverse | 5’- CGTCAATGTTCTGGGTGATG-3’ |
| *Gapdh* | forward | 5’- AAATGGTGAAGGTCGGTGTGAAC -3’ |
|  | reverse | 5’- CAACAATCTCCACTTTGCCACTG -3’ |

Table S4 The antibodies for Western blot and immunofluorescence staining.

| **Antibody** | **Use** | **Use Supplier** | **Cat. No.** |
| --- | --- | --- | --- |
| α-actinin | 1:200 | Abcam | Ab9465 |
| Ki67 | 1:200 | Abcam | Ab16667 |
| Aurora B | 1:500 | Abcam | Ab2254 |
| pH3 | 1:1000 | Millipore | 06-570 |
| 8OHG | 1:500 | Abcam | Ab62623 |
| pATM | 1:500 | Cell Signaling Technology | 4526 |
| donkey anti-rabbit Alexa Fluor 488 | 1:400 | Invitrogen | A-32731 |
| donkey anti-mouse Alexa Fluor 594 | 1:400 | Invitrogen | A-32742 |
| SIRT4 | 1:1000 | Abcam | 124521 |
| SIRT5 | 1:1000 | Proteintech | 15122-1-AP |
| HRP-labeled goat anti-rabbit IgG (H+L) | 1:5000 | ZSGB-BIO | ZB-2301 |
| HRP-labeled goat anti-mouse IgG | 1:5000 | ZSGB-BIO | ZB-2305 |
| WGA | 1:200 | Invitrogen | W32466 |
| cTnT | 1:200 | DSHB | CT3-Supernatant |
